# Levels, Predictors, and Distribution of Interpersonal Solidarity during the COVID-19 Pandemic

**DOI:** 10.3390/ijerph19042041

**Published:** 2022-02-11

**Authors:** Theodor Kaup, Adam Schweda, Julia Krakowczyk, Hannah Dinse, Eva-Maria Skoda, Martin Teufel, Alexander Bäuerle

**Affiliations:** 1 Clinic for Psychosomatic Medicine and Psychotherapy, LVR-University Hospital Essen, University of Duisburg-Essen, 45147 Essen, Germany; theodor.kaup@stud.uni-due.de (T.K.); adam.schweda@lvr.de (A.S.); julia.krakowczyk@lvr.de (J.K.); hannah.dinse@lvr.de (H.D.); eva-maria.skoda@uni-due.de (E.-M.S.); alexander.baeuerle@uni-due.de (A.B.); 2 Center for Translational Neuro- and Behavioral Sciences (C-TNBS), University of Duisburg-Essen, 45147 Essen, Germany

**Keywords:** COVID-19, solidarity, interpersonal solidarity, lockdowns, depression symptoms, mental health

## Abstract

Since introducing the first non-pharmaceutical interventions (NPIs) to decelerate the spread of the virus, European governments have highlighted the role of “solidarity”. However, the role and levels of solidarity, especially during the past lockdowns, is uncertain. The present study thus explores the levels, the role, and the distribution of received and demonstrated interpersonal solidarity during the COVID-19 pandemic. This pooled cross-sectional study was conducted from March 2020 to March 2021 in Germany, including 19,977 participants. Levels of solidarity between the first and the second lockdowns in Germany were compared, possible predictors were examined, and three clusters were defined to unveil distributional patterns of solidarity reception and/or demonstration. To compare solidarity levels between the first and the second lockdowns in Germany, a dummy-coded lockdown variable was introduced and regressed on the two solidarity items. To identify predictors of received and demonstrated solidarity, two multiple linear regression models were computed, testing several demographic and psychological factors. For further exploratory analyses, clusters of “helpers”, “non-helpers”, and “help-receivers and helpers” were computed based on a k-means cluster analysis. Results revealed a lower level of solidarity during the second lockdown compared with the first one. Demonstrated solidarity was positively predicted by adherent safety behavior to avoid COVID-19 infection and by middle age, and negatively by depression symptoms, male gender, and high age. Received solidarity was positively predicted by higher age, by both adherent and dysfunctional safety behavior in avoidance of COVID-19 infection, and by lower educational level. “Helpers” reported little received solidarity but demonstrated high solidarity, “non-helpers” showed both little demonstrated and received solidarity, and “help-receivers and helpers” showed middle–high received and demonstrated solidarity. The three clusters differed the most regarding the variables of age, adherent and dysfunctional safety behavior, fear of COVID-19, subjective risk perceptions regarding contraction of COVID-19 and the respective consequences, and trust in governmental interventions in response to COVID-19. The decrease in interpersonal solidarity over the course of the COVID-19 pandemic, as well as its predictors, should be considered regarding prospective impositions. Furthermore, as depressive symptoms were identified to negatively predict interpersonal solidarity, the adequate provision of mental health services, especially during the COVID-19 pandemic, becomes even more important.

## 1. Introduction

Since the outbreak of the COVID-19 pandemic in 2020, many countries around the world have continued to pursue endeavors linked to preventing the dissemination of the virus, especially through full or partial public “lockdowns.” Various non-pharmaceutical interventions (NPIs), such as contact bans, curfews, and other interventions that aim to stop the spread of the virus, with exception of vaccines or medications, were applied in Germany and other countries [1]. These governmental impositions have been accompanied by frequent appeals to the German population, outlining “solidarity” as a key factor in overcoming the pandemic and the imposed restrictions during the lockdowns [2,3,4]. As these measures and the related solidarity play important roles in the contemporary COVID-19 pandemic, it is important to clarify what solidarity means, what it is based on, and what its contribution is. Prainsack and Buyx outlined solidarity as “enacted commitments to accept costs to assist others with whom a person or persons recognize similarity in a relevant respect” [5] (p. 43). In this regard, they defined three “tiers” of solidarity: institutional, group, and interpersonal solidarity [5] (p. 54–57). Institutional solidarity is based on institutionalized sectors of solidarity, such as welfare programs or social security, while group solidarity, such as wearing masks, contributes to a bilateral benefit and is often associated with socially desired behavior [6]. Interpersonal solidarity occurs on the interpersonal level and is grounded in the shared recognition of the misery or predicament of one that the other can empathize with [7], resulting in grocery shopping for a neighbor in quarantine, for example.

All these forms of solidarity can play a key role in overcoming major crisis events, such as pandemics [8]. For example, evolving high levels of predominantly interpersonal solidarity helped survivors of the Typhoon Haiyan (Philippines) catastrophe in 2013 to organize fast and unconditional relief actions [9]. Moreover, during an extraordinary heat wave in Chicago in 1995, high-level and structured interpersonal solidarity within the Latino community emerged to downsize the mortality rate of elders [10]. Within the contemporary COVID-19 pandemic, an association between higher levels of interpersonal solidarity and fewer reported COVID-19 cases has been assumed [11].

Judging from its levels during the COVID-19 pandemic, interpersonal solidarity appears to be a dynamic trait; according to data from the panel study Austrian Corona Panel Project 2020, solidarity has been continuously waning from the first restrictions of the COVID-19 pandemic from March 2020 to June 2021 [12]. Additionally, the prevailing sentiment of common interpersonal solidarity has shifted toward a dichotomous perception of solidarity, where people have started to compare their own demonstrated solidarity with the received solidarity actions of others, according to interviews from the qualitative panel study SolPan [13]; for example, one participant asked: “Why should I stay at home when I see old people jogging in the street?” [14] (p. 128). For this reason, the present study investigates the two dimensions of interpersonal solidarity—“received solidarity” and “demonstrated solidarity”—separately, by asking if the participant received more help or assistance than usual from others during the previous two weeks, and if the participant themselves provided more help or assistance than usual to others in the previous two weeks.

When examining the role of interpersonal solidarity in the face of the COVID-19 pandemic, past research has suggested that interpersonal solidarity is related to several other factors affected by the COVID-19 pandemic and its repercussions. Hellmann et al. concluded that, among other factors, the perceived level of responsibility toward others and the perceived vulnerability to COVID-19 were positively associated with prosocial behavior [15]. In this respect, the assignation of responsibility to help to others, such as the politicians’ responsibility to help, predicted an increase in prosocial behavior of the individual assigning this responsibility. Marsh et al. assumed that the identification of a fearful facial expression positively predicted prosocial behavior [16], which—although the respective experiments took place in 2007, before the COVID-19 pandemic—can be relevant in light of the current, broadly introduced requirement of mask wearing [17]. However, there is also a lack of research on how certain mental health impairments, such as depression symptoms, general anxiety, and distress, are associated with interpersonal solidarity.

Even though research investigating the level of interpersonal solidarity during the COVID-19 pandemic in Europe exists [12,13,14,18,19], with several studies suggesting an initial peak at the beginning of the pandemic in Germany [15,19], we still lack research on how the level of interpersonal solidarity during the COVID-19 pandemic has been reflected during the second lockdown (which was not only introduced in Germany but analogously in most other European countries in winter 2020). Hence, we assessed the levels of interpersonal solidarity during the first (from March 2020 to May 2020) and the second (from November 2020 to May 2021) lockdowns in Germany. Based on previous research [20], we hypothesized that the level of interpersonal solidarity in the second lockdown was lower than in the first lockdown. Additionally, this study aims to identify possible demographic and psychological predictors of received and demonstrated solidarity. As politics outline the key role of interpersonal solidarity amidst the COVID-19 pandemic, it appears important to know if there are other factors associated with interpersonal solidarity that should hence be recognized and considered for further research. Over the course of the pandemic, several studies have concurrently suggested the increased prevalence of multiple mental health impairments, such as general anxiety, depression symptoms, and stress, in Germany [21,22]. Given the observation that people with depression symptoms often show fatigue or low levels of activity in general [23], we likewise hypothesized that depression symptoms are a negative predictor for demonstrated solidarity, as solidarity demonstration always means “active engagement” [7], and thus requires energy. Additionally, with respect to the observation of the Austrian Corona Panel Project 2020, which saw a concurrent decrease in perceived solidarity and satisfaction with the government regarding COVID-19, we hypothesized that trust in governmental interventions regarding COVID-19 is related to received solidarity. Last, but not least, our third hypothesis was that older ages are positively correlated with received solidarity, as elders were declared a “risk group” of high concern by the German government, from the beginning of the pandemic onward—a group at whom solidarity should primarily be directed [2]. Finally, concerning reports from the panel study SolPan that suggested a dichotomous perception of interpersonal solidarity after the beginning of the pandemic, we explored the distribution of received to demonstrated solidarity, and thereupon identified three clusters (“helpers”, “non-helpers”, and “help-receivers or helpers”) within this distribution. In this context, we looked into how the investigated demographic and psychological factors may also have contributed to the distribution of received and demonstrated solidarity within this cluster display.

## 2. Materials and Methods

### 2.1. Sample Description

The final sample consisted of 19,977 participants. Of those, 33% identified as male, 66% identified as female, and 1% identified as diverse. Of all the participants, 3075 (15.4%) were aged 18–24 years, 4757 (23.8%) were 25–34 years, 4464 (22.3%) were 35–44 years, 3808 (19.1%) were 45–54 years, 2821 (14.1%) were 55–64 years, 912 (4.6%) were 65–74 years, and 141 (0.7%) were 75 or above. A total of 2959 (14.8%) participants reported a diagnosed mental illness. Inclusion criteria for participating were adequate knowledge of the German language and age above 18. For a detailed description of the demographic characteristics, see Table 1.

### 2.2. Study Design and Procedure

This pooled cross-sectional study used data from an online, web-enabled survey, undertaken from March 2020 to March 2021. Recruitment took place on online and social media platforms, such as Facebook, as well as via TV, radio, and newspaper reports. The participation in the study was on voluntary basis and did not use any incentives or rewards. The survey included 27,092 participants in total, of which 19,977 completed the survey (completion rate = 73.7%); the mean completion time was 12 min and 18.64 s. Only complete cases were examined further, incomplete questionnaires were eliminated. For the analysis of differences between the 1st and 2nd national lockdowns, only cases collected between 23 March 2020 and 5 April 2020, comprising 1513 participants, and between 16 December 2020 and 14 January 2021, with 841 participants, were analyzed. Electronic informed consent was given by all participants. The execution of the study was approved by the Ethics Committee of the Medical Faculty of the University of Duisburg-Essen (20-9307-BO).

### 2.3. Materials

To assess the current level of interpersonal solidarity, the assessment used 2 items, utilizing 7-point Likert scales from 1 = “I do not agree at all” to 7 = “I totally agree”. The first item asked if one had received more help or assistance than usual from others in the previous two weeks (“*received solidarity*“), while the other asked if the participant themselves had provided more help or assistance than usual to others in the previous two weeks (“*demonstrated solidarity”*).

As part of the multiple regression models, the survey assessed basic demographic criteria, such as age, gender, occupational situation, educational degree, marital status, and home community size. Furthermore, it involved various validated instruments, estimating the participants’ present mental health status and safety behavior during the pandemic. Generalized anxiety symptoms were assessed via the General Anxiety Disorder 7 (GAD-7) screening tool, using a 4-point Likert scale (from 0 = “not at all” to 3 = “nearly every day” [24,25]), with an internal consistency of α = 0.89 [25]. Depression symptoms were measured via the Patient Health Questionnaire 2 (PHQ-2), using a 4-point Likert scale (from 0 = “never” to 3 = “nearly every day” [26,27]), with 2 items screening the frequency of depression symptoms over the previous 2 weeks, yielding an internal consistency of α = 0.83 [27]. Distress was assessed via the Distress Thermometer (DT) (analog scale from 0 = “no distress” to 10 = “extreme distress” [28,29]).

To evaluate safety behavior during the pandemic, the survey distinguished between *adherent safety behavior* (ASB) and *dysfunctional safety behavior* (DSB). There were 4 items generated for each (using a 7-point Likert scale, ranging from 1 = “strongly disagree” to 7 = “strongly agree”), to assess either recommended safety manners (in accordance with the WHO’s recommendations [30]) or inappropriate/excessive changes in safety behavior. The 4 ASB-defining items showed an internal consistency of α = 0.738, and the DSB-defining items reached an internal consistency of α = 0.770 [31]. For further information, see Supplementary Materials.

*Fear of COVID-19* was assessed by one item (“I worry about COVID-19”), using a 7-point Likert scale from 1 = “strongly disagree” to 7 = “strongly agree”. Another 5 independent items assessed the subjective risk of a possible infection with COVID-19 and its outcome (“How high do you estimate the risk of: %-*Risk_infection_* = Getting infected with COVID-19?; %-*Risk_symptoms_* = evolving symptoms?; %-*Risk_severe_* = a severe progress?; %-*Risk_dying_* = dying?; %-*Risk_transmit_* = transmitting the virus?”), measured by presenting a digital scale for self-assessment to measure the estimated risk from 0 to 100% by means of a slider control.

*Trust in governmental interventions in response to COVID-19* (“I think Germany is well prepared to face COVID-19; I think all government measures are being taken to combat COVID-19; I have confidence in the governmental system in Germany”) and the *subjective level of information concerning COVID-19* (“I feel informed about COVID-19; I feel informed about measures to avoid an infection with COVID-19; I understand the health authorities’ advice regarding COVID-19”) were assessed using a 7-point Likert scale (from 1 = complete disagreement to 7 = complete agreement). Both scales showed high internal consistency with Cronbach’s *α = 0.825 and Cronbach’s α =* 0.801, respectively [21].

### 2.4. Data Analysis

The acquired data were analyzed using SPSS Statistics Software 27 (IBM, Armonk, NY, USA) and R 3.6.0. (RStudio Team) [32]. First, the mean solidarity scores of received and demonstrated solidarity during the first and second national lockdowns were measured and thereupon compared. To do so, the dummy-coded lockdown variable was regressed on the solidarity items, also including gender, age, community size, education, and presence of a diagnosed mental disorder as covariates. The given data showed neither normal distribution characteristics nor equal variances regarding the variables of interest, as computed by Kolmogorov–Smirnov and Breusch–Pagan Tests. Yet, robust regression models using Huber–White standard errors yielded equivalent results. Subsequent marginal effects were computed to allow for a quantification of effect sizes. For interpretation of these, we followed the recommendations by Cohen [33].

The subsequent regression models of the predictors of solidarity were conditioned on age, educational level, gender, community size, and mental illnesses. As part of the regression analyses, all given data from the 19,977 completed cases provided by the survey from March 2020 to March 2021 were applied. Hence, to determine the conditional individual contributions of the variables on interpersonal received and demonstrated solidarity, two standard linear regression models were applied on both received and demonstrated solidarity. Yet, after implementation, violations in normality of residuals and homoskedasticity became apparent. Still, models with robust Huber–White standard errors (see Supplementary Materials Table S8) yielded almost identical results, so a lack of normality and homoscedasticity did not compromise our results. Multicollinearity was found irrelevant, as none of the variables’ variance inflation factors exceeded a value of 10 [34]. For each model, the coefficient of determination R^2^ was reported, assuming values above 0.02 as mild, above 0.13 as moderate, and above 0.26 as strong goodness of fit [35]. As extensive sample sizes can quickly reach significant *p*-values and lead to rejecting the null hypothesis [36], the respective size of the regression coefficient, as well as the confidence intervals, were also considered when interpreting our results. In order to explore distributive patterns and quantitative differences between the two interpersonal solidarity dimensions, the scores of received and demonstrated solidarity were related to each other and then clustered by k-means into three cluster groups of “helpers”, “non-helpers”, and “help-receivers and helpers”. “Helpers” reported having demonstrated significant solidarity but, on the other hand, having received only little solidarity, “non-helpers” reported both little received and demonstrated solidarity, while “help-receivers and helpers” received medium solidarity and demonstrated medium–high solidarity. The quantity of clusters was estimated via gap statistic [37]. In order to further characterize the clusters in terms of their sociodemographics, their attitudes toward COVID-19-related topics, and their mental health, χ²-tests and regression analyses were applied.

## 3. Results

**Figure 1:**
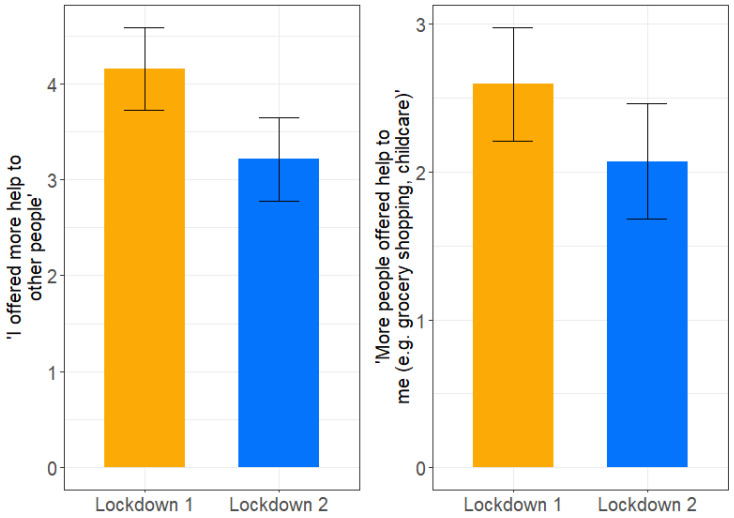
Alternations of received and demonstrated solidarity between the periods of lockdown 1 (*n* = 1513) and lockdown 2 (*n* = 841). The bar charts show marginal means of the linear regression model also including gender, age, community size, education, and presence of a mental illness as covariates. Error bars show 95% confidence intervals. Note: mean valued standard errors +/−1 as error bars of received solidarity (“More people offered help to me”, e.g., grocery shopping, childcare) and demonstrated solidarity (“I offered more help to other people”) during lockdown 1 and lockdown 2.

Significant differences between the mean scores for both interpersonal solidarity dimensions between the first and the second lockdowns were found. Both levels of received and demonstrated solidarity were significantly lower during the second lockdown than during the first lockdown (see Figure 1). *Demonstrated solidarity* provided the highest decrease from the first lockdown to the second lockdown (mean difference in marginal effects (*demonstrated solidarity*) = 0.946, *t* (2335) = 11.622, *p* < 0.0001, *95% CI* (0.786; 1.11), *d* = 0.521), with a moderate effect. Additionally, *received solidarity* significantly decreased from the first to the second lockdown with a small effect (mean difference in marginal effects (*received solidarity*) = 0.523, *t* (2335) = 7.193, *p* < 0.0001, *95% CI* (0.381; 0.666), *d* = 0.323).

### 3.1. Exploration of Predictors

The results of the two multiple regression models with the dependent variables—*received solidarity* and *demonstrated solidarity*—as well as variable-wise F-tests, can be found in the Supplementary Materials (Tables S1 and S2). The first model, with *demonstrated solidarity* as a dependent variable, reached an adjusted R^2^ of 0.130; the second model, including *received solidarity* as the outcome, reached an adjusted R^2^ of 0.095. Model 1 provided a moderate goodness of fit, and model 2 a mild goodness of fit. In the following, only global variable-wise F-tests will be reported. All marginal effects and post hoc contrasts can be found in the Supplementary Materials (Tables S3—S7).

As part of the first regression model, with *demonstrated solidarity* as the dependent variable, the model identified four dominant independent variables: depression symptoms, gender, age, and adherent safety behavior. *Demonstrated solidarity* was negatively predicted by *depression symptoms (F (1, 19941) = 116.1258, p < 0.001). Male* gender negatively predicted demonstrated solidarity *(F (2, 19941) = 51.6973, p < 0.001).* Positive predictors for demonstrated solidarity, on the other hand, were the ages between younger and middle age *(F (6, 19941) = 11.8937, p < 0.001),* and *ASB (F (1, 19941) = 833.6286, p < 0.001).*

Concerning the second regression model, with *received solidarity* as the dependent variable, five dominant significant predictors could be determined: age, educational degree, marital status, and adherent and dysfunctional safety behavior. Here, ages from 25 onward positively predicted received solidarity *(F (6, 19941) = 50.5887, p < 0.001)* with a sharp peak at higher ages—>65 years. Low educational degrees (secondary school or below) were positive predictors *(F (5, 19941) = 7.3600, p < 0.001)*. Participants in a marriage or relationship appeared to be less frequent recipients of solidarity than participants with other marital statuses *(F (5, 19941) = 6.8225, p < 0.001).* Regarding the role of safety behavior, ASB and DSB both positively predicted *received solidarity (F (1, 19941) = 171.2620, p < 0.001; F (1, 19941) = 277.9808, p < 0.001)*.

### 3.2. Cluster Analysis

**Figure 2:**
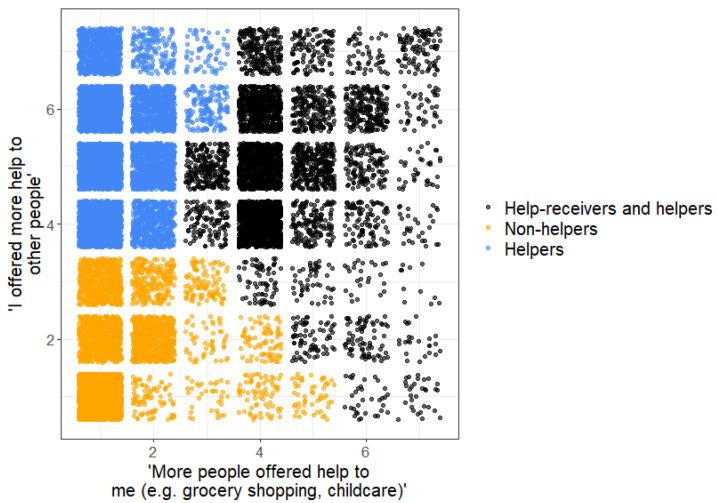
K-means clustered distribution pattern of received (“More people offered help to me”) to demonstrated (“I offered more help to other people”) solidarity.

To explore distributive patterns and quantitative differences between *received* and *demonstrated solidarity*, the scores of the two interpersonal solidarity dimensions were plotted against each other on a grid chart, clustered by k-means, and divided into cluster groups of “helpers”, “non-helpers”, and “help-receivers and helpers” (see Figure 2). The cluster of “helpers” consisted of 5756 participants, the cluster of “non-helpers” consisted of 8998 participants, and the cluster of “help-receivers and helpers” consisted of 5224 participants.

While most cases indicating low *demonstrated solidarity* also reported little *received solidarity* (“non-helpers”), the displayed distribution of “helpers” diverged. Concerning the cluster of “helpers”, a concentration of reported cases showing moderate–high *demonstrated solidarity* but low *received solidarity* was found. The third cluster of “help-receivers or helpers” showed moderate *received solidarity* and moderate–high *demonstrated solidarity*. The initial and final cluster centers can be seen in Table 2.

Changes in the cluster’s centroids yielded 0 in the 4th iteration. A comparison of the mean values of all the demographic variables that were included in the previous regression models can be found in the Supplementary Materials (Table S9). These comparisons revealed that the “help-receivers and helpers” cluster included individuals who tended to be older. Individuals in the “non-helpers” cluster most prominently differed in that they exhibited comparably low levels of fear of COVID-19 and both adherent and dysfunctional safety behavior, as can be seen in the Supplementary Materials (Figures S4 and S5). Similarly, they regarded the risk of contracting COVID-19 and its respective consequences as lower than individuals in the “helpers” and “help-receivers and helpers” clusters (see Supplementary Materials Figure S6).

## 4. Discussion

In this study, we assessed and compared the levels of received and demonstrated solidarity during the first and second national lockdowns in Germany. Furthermore, we aimed to identify predictors of received and demonstrated solidarity. Finally, the objective of this study was to explore the distribution of these two interpersonal solidarity dimensions. Interpersonal solidarity was found to be lower in the second lockdown, compared with the first. Demonstrated solidarity was positively predicted by the adherent safety behavior of avoiding COVID-19 infection (ASB) and by ages from 35 to 64 years, and negatively predicted by depression symptoms, male gender, and high age; whereas, received solidarity was positively predicted by higher age, adherent and dysfunctional safety behavior (DSB) of avoiding COVID-19 infection, and lower educational levels. Moreover, received and demonstrated solidarity were not equally distributed but showed accumulations of participants that reported having demonstrated significant solidarity but, on the other hand, having received only little solidarity (“helpers”), participants that reported both little received and demonstrated solidarity (“non-helpers”), and participants having received medium solidarity and demonstrated medium–high solidarity (“help-receivers and helpers”). The differences between these three cluster groups were mostly determined by age, safety behavior, fear of COVID-19, and the perceived subjective risk regarding contraction of COVID-19 and the respective consequences, and the trust in governmental interventions in response to COVID-19.

Regarding the development of interpersonal solidarity in other European countries, various studies generally suggested a decrease in interpersonal solidarity over the course of the pandemic [12,20]. In Germany, studies suggested that demonstrated solidarity initially peaked in terms of new help arrangements [19] and increased prosocial behavior [15] in April 2020; whereas, the levels of interpersonal solidarity during the second lockdown were uncertain. In the present study, both received and demonstrated solidarity were lower during the second lockdown compared with the first lockdown, supporting the initial hypothesis. The roots of this observation are surely many, but several possible reasons for this waning are part of the ongoing discussion. Concerning the surge of solidarity in the Netherlands between March and October 2020, referring to qualitative data from the SolPan study, the authors assumed that this phenomenon, on one hand, could be explained by a stepwise normalization of the threat over time [21]; on the other hand, they concluded that the lack of a contribution from institutional carriers of solidarity and, thus, the delegation of all responsibility to maintain solidarity to individual citizens, could have yielded the decrease in solidarity as well. This assumption found further recognition regarding the evaluation of interviews in the neighboring state Belgium, where, in contrast to the Dutch example, the trust in strong social security and public healthcare systems seemed to foster the adherence to COVID-19 restrictions and maintenance of solidarity [38].

Regarding possible predictors of interpersonal solidarity, the given results were in line with our hypothesis of depression symptoms being negatively associated with demonstrated solidarity, owing to a reduced level of activity that active interpersonal solidarity presupposes. Concerning our second hypothesis, a relevant positive association between the trust in governmental interventions in response to COVID-19 and received solidarity could not be finally confirmed, as the effect was only minuscule (see Supplementary Materials Table S1). Consistent with our third hypothesis, high age was positively associated with received solidarity, as we assumed that older people needed more help and assistance than younger people, and additionally were declared by the government to be a “risk-group” amidst the pandemic, whom solidarity demonstration should primarily be addressed to [2]. Middle age (35–64), on the other hand, showed the most demonstrated solidarity of all age groups, which was in line with past research [19]. When evaluating possible reasons for this, we assumed that middle age groups usually have the most familial responsibility for both children and parents, which also requires the most demonstrated solidarity (although, this question remains for further research). Koos and Bertogg also discovered, in this regard, that the most solidarity was demonstrated among family members [19].

A negative predictor for demonstrated solidarity was male gender. One possible explanation could be the overrepresentation of females among the health and home care sector [39], which leads to a certain confounding of our results, as the survey did not specify the context in which the demonstrated solidarity was provided. When evaluating the role of safety behavior, ASB positively predicted both demonstrated and received solidarity, and DSB positively predicted received solidarity. One possible explanation for the positive relation between ASB and demonstrated solidarity is that safety behavior, such as washing hands or avoiding public spaces, could be understood as a demonstration of solidarity in terms of minimizing the risk of transmitting the virus to others, and thus may have intersecting dimensions with solidarity demonstration. On the other hand, concerning the positive relation of ASB/DSB and received solidarity, it is important to mention that either form of safety behavior was higher in age groups 55+ compared with those below 55, which is in line with previous research [31]. The observed associations between ASB/DSB and receiving solidarity were thus at least partially biased by the impact of age, as older participants not only showed higher safety behavior but also received more solidarity. Low educational degree positively predicted received solidarity as well. One possible explanation is that people with lower degrees of educational were more likely to be affected by unemployment and layoffs [40] and be in need of solidarity of all kinds. However, the role of educational degree regarding interpersonal solidarity, especially in the group of children and adolescents, should be investigated by further research on facing the many disruptions in the academic sector due to measurements to contain the spread of COVID-19.

Additionally, reports from the Austrian Panel Project 2020 suggested a dichotomous perception of interpersonal solidarity, which subdivided it into “received” and “demonstrated” solidarity. In this context, our second observation when assessing the level of interpersonal solidarity during the German lockdowns was that, in both cases, demonstrated solidarity appeared higher than received solidarity, an observation that recent studies on received and demonstrated solidarity during the COVID-19 pandemic in Germany could make as well [19]. When exploring the distribution of received and demonstrated solidarity in this regard, it appeared that this discrepancy did not show a consistently distributed pattern where all participants generally reported having received less solidarity than they had demonstrated. Rather, we identified three groups, as follows: “helpers”, that demonstrated significant solidarity but reported only little received solidarity; “non-helpers”, that reported just as little received as demonstrated solidarity; “help-receivers and helpers”, that reported middle received and middle–high demonstrated solidarity. The biggest differences between these three clusters were related to their characteristics in terms of age, safety behavior, trust in governmental interventions in response to COVID-19, fear of COVID-19, and the perceived subjective risk of contracting the virus with the respective possible consequences.

“Helpers” were the biggest proportion of participants aged 35–54 years. In the context of the previous finding that middle-aged participants showed most demonstrated solidarity, compared with all other age groups, the composition of the “helpers” cluster seemed to be in line with this finding. The “non-helpers”, on the other hand, were the largest proportion of participants between 25–34 years. Additionally, they showed the least safety behavior (ASB and DSB), the lowest fear of COVID-19, the lowest perceived risk of contracting COVID-19 with the respective possible consequences, and the lowest trust in governmental interventions in response to COVID-19. Concerning safety behavior, this also appeared to be in line with the study’s findings that older participants showed more safety behavior than younger participants, and vice versa—younger participants showed less. In this regard, the described association between fear of COVID-19 and safety behavior [31] may have also mattered in terms of ignoring safety behavior, if one’s perceived fear of COVID-19 was only minor.

In view of the low reported fear of COVID-19, recent studies have equally discovered that younger age groups showed low fear (as it can be assumed, as younger age groups have a smaller likelihood of developing a severe case of COVID-19 [21]). Accordingly, this could also explain why the perceived subjective risk regarding contracting COVID-19, developing symptoms, severe progress, or dying, was the lowest in this cluster, too. In terms of which aspects are compromised by the reported fear of COVID-19, studies offer a wider range of response options to describe fear that relate to a broader context outside of health-related issues, such as worries about the future, fear of contact bans, fear of losing relatives with preexisting health conditions, or the risk of progressing to a severe course in case of infection [41]. Another characteristic of the “non-helpers” cluster was the low level of trust in governmental interventions in response to COVID-19. Although we could not confirm our second initial hypothesis that the trust in governmental interventions in response to COVID-19 was related to received solidarity within the regression analysis, the fact that “non-helpers” showed the lowest trust of all three clusters implied that the effective impact of this variable may still have a certain relevance. Younger people in this cluster were especially affected by the restrictive governmental interventions, which mostly included school closures and contact bans that were suspected of having led to increasing psychological symptoms and thus to a loss of trust in governmental interventions [42]. Furthermore, the isolation that resulted from these interventions logically may have complicated the possibility to receive or demonstrate interpersonal solidarity, and thus explain why interpersonal solidarity was found the lowest in this cluster, in contrast to the predominantly middle-aged “helpers” in the age range of 35–54 years, that were demonstrating more solidarity, as they could have had more opportunities to receive and demonstrate solidarity from family members and their partner. The third cluster, “help-receivers and helpers”, showed the biggest proportion of participants aged 55 years or above. In the case of this cluster, in contrast to the “non-helpers” cluster, safety behavior (ASB and DSB), fear of COVID-19, and the perceived subjective risk regarding contracting COVID-19 and the respective consequences were the highest of all three clusters. The age structure of this cluster was in line with our previously confirmed hypothesis that older ages are positively associated with receiving solidarity. On top of that, the finding that ASB and DSB were higher in the age groups aged 55+ years seemed to be true for this cluster as well. In this context, the fact that the fear of COVID-19 and the perceived subjective risk regarding contraction of COVID-19 and the respective consequences were also the highest in this cluster could be related to the higher age of the respective participants, as older people are more likely to develop severe illness in cases of infection than younger people. Again, the association between fear of COVID-19 and safety behavior [31] may have played a role at this point, as this fear (and the perceived subjective risk of contracting COVID-19, developing a severe progress, or even dying) may have led to higher safety behavior.

To sum up, the present study revealed the level of interpersonal solidarity in Germany during the first and second national lockdowns, as well as its predictors, such as depression symptoms, safety behavior, age, and educational degree. Additionally, the suggested impression of a dichotomous perception of interpersonal solidarity [14] could be diversified within our cluster analysis into three separate groups based on the distributions of received and demonstrated solidarity, clarifying the roles of age, safety behavior, the fear of COVID-19, and trust in governmental interventions in response to COVID-19, in the context of how these factors may be situated within the present perception of interpersonal solidarity.

As interpersonal solidarity during the second lockdown was lower than in the first lockdown, with a simultaneously elevated mental health burden among the population, the apparent association between depressive symptoms and demonstrated solidarity supports the assumption that upcoming lockdowns will aggravate the decrease in interpersonal solidarity even more. Hence, to maintain high levels of interpersonal solidarity, the sufficient and adequate provision of health care in the COVID-19 pandemic appears even more important. In this regard, the support of institutional carriers of interpersonal solidarity should not be disrupted at this later stage of the pandemic, as delegating all responsibility to act in solidarity to the individual was assumed to have led to an expiration of interpersonal solidarity in other European countries [20]. Contrariwise, if institutional solidary structures are maintained, it should be prevented that solidarity on individual level declines. Therefore, educational programs that implement the role of each tier of solidarity should be introduced to empower individuals to overcome major crisis events, such as the contemporary pandemic, by maintaining high levels of solidarity. In this concern, the role of age especially should receive further attention. While middle-aged people between 35 and 54 years were most likely to demonstrate solidarity, young adolescents in particular showed comparatively low levels of solidarity demonstration, with low levels of COVID-19-related fear, safety behavior, and trust in the governmental interventions. In this regard, further measurements to contain the spread of the virus should also incorporate youths and young adolescents, foster their mental health, promote safety behavior, and gain compliance for prospective interventions in response to COVID-19, in order to establish higher levels of interpersonal solidarity in this age group as well. Again, one possibility of doing so could be to initiate solidarity promotion campaigns adapted to this specific age group, and low-threshold provision of accessible information in schools, universities, and other community facilities. These interventions could be useful to support the role of solidarity of younger people in times of a pandemic, provide the opportunity to talk, and to raise COVID-19-related fears, misinformation, depression symptoms, and other reasons that may cause young people to act less solidary. In this concern, the German Expert Council on COVID-19 recommended a corresponding, age-adapted, target-group-orientated approach for health and risk communication as well [43]. However, an approach like this predisposes interpersonal contact, which can act as a barrier due to possible contact restrictions during the COVID-19 pandemic. The present study also investigated the relation of an educational degree to interpersonal solidarity. Nonetheless, the impact of education on interpersonal solidarity should be further researched, especially in light of the interruption in or obstruction of the psychosocial development of children from several COVID-19-related restrictions that are assumed to be related to this development [17,44]. Furthermore, as this study derived its data mainly from 2020 and early 2021, further research is needed to monitor the continued development of interpersonal solidarity over the course of the pandemic. This concerns, in particular, the consideration of factors that the present study could not yet imply, such as the influence of the COVID-19 vaccines that were introduced by the end of 2020. While in the beginning of the pandemic, the term “solidarity” was mainly raised to gain adherence for the respective measures to protect vulnerable groups, such as elders, officials also promote the vaccination on the background of solidary behavior to protect others [45]. As solidarity, according to Prainsack and Buyx, can be understood as “enacted commitments to accept costs to assist others with whom a person or persons recognize similarity in a relevant respect” [5] (p. 43), solidary behavior predisposes the possibility to assist or help others. In case of the current vaccinations against COVID-19, the relevance for increasing solidarity by means of protecting others, however, is not indisputably proven [46,47]. Hence, further research should also investigate the development of solidarity in relation to the current vaccination campaigns in Europe. In this respect, the impact of the 2021 ongoing vaccination campaigns in Europe may already be especially relevant for the development of solidarity, as the governmental rhetoric about people who hesitate to be vaccinated may have an equally negative impact on interpersonal solidarity as the rhetoric on “risk-groups” may have had at the beginning of the pandemic in 2020, by enhancing “the perception of differences between groups” and “animosities” [14] (p. 129), rather than similarities, amidst the COVID-19 pandemic.

The present study is one of the biggest studies so far assessing interpersonal solidarity in Europe, as measured by its sample size of 19,977 participants. Nevertheless, a number of limitations need to be considered when interpreting the results. When measuring demonstrated and received solidarity, the tendency of participants to evaluate their demonstration of solidarity higher than what they receive could be linked to the overconfidence bias [48], which accompanies constant over-placement of one’s abilities relative to others [49]. At the same time, as demonstrating solidarity in times of crisis displays a socially desired behavior, the social desirability effect bias [50] may also have had an influence on the reported demonstrated solidarity. We could identify depression symptoms to negatively predict solidary demonstration. However, as our hypothesis was that depression symptoms predicted demonstrated solidarity due to the reduced level of activity that can be seen in depressive persons, this study cannot finally clarify if—and if so, to what extent—this factor or others that are described as depressive symptoms may have played a role, concerning the relation between depressive symptoms and solidary demonstration. Further research is required in this regard to investigate whether various individual depressive symptoms, such as insomnia, reduced concentration, or the reduced level of activity, may contribute in particular to the observed relation between depression symptoms and demonstrated solidarity. The chosen subdivision of interpersonal solidarity in demonstrated and received solidarity was applied with respect to reports from the Austrian Panel Project 2020, suggesting a dichotomous perception of solidarity. Under this circumstance, interpersonal solidarity was assessed with only two items, which could decrease the signal-to-noise ratio, compared with using an entire, validated psychometric instrument. Furthermore, we chose to contextualize these two solidarity items with the concept of solidarity by Prainsack and Buyx [5], as it considers several historical and multidisciplinary perspectives and thus offers a universal approach, which can be adapted across different disciplines. Nonetheless, there is great intellectual discussion on whether, and to what extent, terms such as fraternity, pro-social behavior, or altruism cover intersections of the solidarity concept, and how they must be illuminated in a given historical context [51]. Hence, comparisons between studies issuing solidarity should be carried out with caution. As this online survey used collected data distributed via online and analog channels, a possible selection bias should be taken into account. An overrepresentation of women among the participants could also be connected to this bias. Furthermore, the survey did not collect information about nationality or ethnicity. As we used the k-means clustering algorithm, it is important to mention that the use of an algorithm that divides the population into a predefined quantity of discrete clusters may not be realistic. Hence, alternative clustering techniques that find a probabilistic approach for cluster structure may provide supplemental information [52].

## 5. Conclusions

This study aimed to investigate the level of interpersonal solidarity in Germany during the COVID-19-related lockdowns, identify possible predictors for interpersonal solidarity, and explore the distribution of received and demonstrated solidarity. A decrease in interpersonal solidarity during the second lockdown in Germany was discovered, compared with the first lockdown. Among others, depression symptoms, age, and adherent safety behavior were the most relevant predictors of interpersonal solidarity. Furthermore, received and demonstrated solidarity were not equally distributed, but showed three accumulations within the distribution that differed regarding mainly their age structure, safety behavior, fear of COVID-19, and trust in governmental interventions in response to COVID-19. Although political leaders highlighted the utmost importance of maintaining solidarity when facing the second national lockdown in October 2020, our findings suggested a depletion of interpersonal solidarity. To maintain high interpersonal solidarity, other approaches that also emphasize factors associated with interpersonal solidarity should be taken into account, not only in regard to the ongoing COVID-19 pandemic and measures against the spread of the virus, but also to other major crises which society may face in the future. This pertains to educational and promotional campaigns issuing the role of solidarity during a crisis, such as a pandemic, that address and empower youths and younger adults especially and emphasize adherent safety behavior, as well as supplying mental health support to tackle depression symptoms. Moreover, to maintain high levels of solidarity, further research and monitoring of solidarity development is essential—especially in regard to the potential impact the vaccination and corresponding attitudes may have on solidarity.

## Figures and Tables

**Table 1:** Demographic characteristics.

Variable	*n* (Total) = 19,977	*n* (Lockdown 1) = 1513	*n* (Lockdown 2) = 841
**Gender** Male Female Diverse	6580 (32.9%) 13,306 (66.6%) 91 (0.5%)	460 (30.4%) 1047 (69.2%) 6 (0.4%)	359 (42.7%) 477 (56.7%) 5 (0.6%)
**Age** 18–24 years 25–34 years 35–44 years 45–54 years 55–64 years 65–74 years Above 75 years	3075 (15.4%) 4757 (23.8%) 4464 (22.3%) 3808 (19.1%) 2821 (14.1%) 912 (4.6%) 141 (0.7%)	154 (10.2%) 356 (23.5%) 356 (23.5%) 315 (20.8%) 238 (15.7%) 82 (5.4%) 12 (0.8%)	172 (20.5%) 221 (26.3%) 177 (21.0%) 149 (17.7%) 90 (10.7%) 24 (2.9%) (1.0%)
**Education** University Degree High School Degree Secondary School Degree First School Degree No School Degree Others	7994 (40.0%) 6427 (32.2%) 4220 (21.1%) 969 (4.8%) 72 (0.4%) 296 (1.5%)	672 (44.4%) 465 (30.7%) 270 (17.8%) 75 (5%) 6 (0.4%) 25 (1.7%)	300 (35.7%) 270 (32.1%) 212 (25.2%) 50 (5.9%) 4 (0.5%) 5 (0.6%)
**Community size** Large city (> 100,000 inh.) Medium-sized city (> 20,000 inh.) Small town (> 5000 inh.) Rural area (< 5000 inh.)	9672 (48.4%) 4694 (23.5%) 2715 (13.6%) 2897 (14.5%)	789 (52.1%) 359 (23.7%) 186 (12.3%) 179 (11.8%)	295 (35.1%) 210 (25.0%) 146 (17.4%) 190 (22.6%)
**Mental illness**	2959 (14,8%)	195 (12.9%)	85 (10.1%)
**Marital status** Single Married In relationship Divorced Widowed Others	5997 (30%) 8234 (41.2%) 4140 (20.7%) 1183 (5.9%) 261 (1.3%) 163 (0.8%)	419 (27.7%) 637 (42.1%) 324 (21.4%) 106 (7.0%) 19 (1.3%) 8 (0.5%)	284 (33.8%) 307 (36.5%) 199 (23.7%) 40 (4.8%) 4 (0.5) 7 (0.8%)

**Table 2:** Initial and final cluster centers of “non-helpers”, “helpers”, and “help-receivers and helpers”.

	Initial Cluster Centers			Final Cluster Centers		
	Non-Helpers	Helpers	Help-Receivers and Helpers	Non-Helpers	Helpers	Help-Receivers and Helpers
received solidarity	4.00	1.00	7.00	1.35	1.32	4.51
demonstrated solidarity	1.00	7.00	7.00	1.59	5.26	4.88

## Data Availability

The data presented in this study are available on request from the corresponding author.

## Supplementary Materials

The following supporting information can be downloaded at: https://www.mdpi.com/article/10.3390/ijerph19042041/s1, Supplementary Material S1: English translation for ASB, DSB, and Trust in governmental interventions in response to COVID-19; Supplementary Material S2: Additional information on the presented regression tables and cluster characterization; Table S1: Regression coefficients of all variables on received and demonstrated solidarity; Table S2: ANOVA tests with received and demonstrated solidarity as dependent variable; Tables S3—S7: marginal effects and post hoc contrasts on received and demonstrated solidarity; Table S8: Regression coefficients of robust regression model; Table S9: Demographic characteristics stratified across the three clusters; Figure S1: Marginal effects of the most dominant effects of predictors regressed on received solidarity; Figure S2: Marginal effects of the most dominant effects of predictors regressed on demonstrated solidarity; Figure S3: Group-wise proportions of participant characteristics; Figure S4: Differences between the clusters in terms of their levels of COVID-19-related fear; Figure S5: Differences between the clusters in terms of their levels of ASB and DSB; Figure S6: Differences between the clusters in terms of their subjective risk assessments as to contracting COVID-19 and its respective consequences.
