# Supplementary material for: Levels, Predictors, and Distribution of Interpersonal Solidarity during the COVID-19 Pandemic
Source: Int J Environ Res Public Health. 2022 Feb 11;19(4):2041. doi: 10.3390/ijerph19042041 (PMC8872525; doi:10.3390/ijerph19042041)
Supplement: Supplementary file 1 [file ijerph-19-02041-s001.zip › ijerph-1563561-supplementary.pdf]

## Supplementary Material S1

Three of the presented scales in the article were self-generated: *adherent safety behavior* (ASB), *dysfunctional safety behavior* (DSB) and *trust in governmental interventions in response to COVID-19.* English translations can be found below.

### Adherent Safety Behavior

- I wash / disinfect my hands more frequent
- I increasingly avoid public places / events
- I increasingly avoid public transport (subway, tram, bus train)
- I have changed my trip / vacation plans or I would change them if I had planned a vacation / trip.

### Dysfunctional Safety Behavior

- I have bought larger quantities of basic food (flour, sugar, noodles, rice, and canned food) or will buy more in the near future.
- I have bought larger quantities of hand disinfection/soap/similar or will buy more in the near future.
- I have bought larger quantities of toilet/hygiene articles or will buy more in the near future
- I have become more selfish in my behavior.

### Trust in governmental interventions in response to COVID-19

- I think all government measures are being taken to combat COVID-19.
- I have confidence in the governmental system in Germany.
- I think Germany is well prepared to face COVID-19.
- I believe that political actions against COVID-19 in Germany are exaggerated.

## Supplementary Material S2

### Regression tables and variable-wise F-tests

In the manuscript, we describe two regression models that illustrate the predictors of either reporting to have received more help or to have offered more help during the COVID-19-pandemic. Here, the reader can find the regression tables (see table S1), as well as variable-wise F-tests that indicate global influences of the respective variable (ANOVA, see table S2). Marginal effects are illustrated in figure 1 and 2. Post-hoc comparisons for the categorical predictors are described in tables S3-S7.

### Robust regression models

In the manuscript, we reported the results of two ordinary-least-squares regression models (see table S1). Yet, according to results from Shapiro-Wilk- and Breusch-Pagan-Tests, there are violations of normality of residuals and homoskedasticity. Therefore, we reproduced the analyses using regression models with robust Huber-White standard errors (see table S8). Indeed, the results are almost identical.

### Characterization of clusters

In the main manuscript, we report results from a cluster analysis that identified three clusters from the joint distributions of participants‘ self-reported demostrated solidarity behavior, as well as the perceived solidarity behavior by others during the COVID-19 pandemic. These analyses revealed three clusters, one which indicated that they did not

exhibit more helping behavior and did not experience any more helping behavior by others („non-helpers“), one which primarily indicated that they indicated more solidarity („helpers“), and one cluster that consisted of individuals who reported having received more help or having received more help and demonstrated more help. For the sake of brevity, the authors decided to add the exact characterization – including demographic characteristics etc. – to the supplemental materials.

Table S9 shows the participant characteristics stratified across the respective clusters. Please notice that, although we used standard $\chi^2$-tests to compare the respective distributions of participant features between the clusters, p-values remain difficult to interpret due to the large sample size. Hence, while all reported $\chi^2$-tests in table S9 show p-values below .01, we only consider some of these differences practically significant. Here, it can be observed that the „help-receivers and helpers“ cluster includes a higher proportion of individuals who are older (see figure S3 – Panel A), as well as a slightly higher proportion of people who report having diseases predisposing to a more severe course of COVID-19 (see figure S3 – Panel B).

Next, we asked whether the clusters differed in terms of their levels of mental symptoms, their attitudes toward COVID-19 specific topics and their behavior during the COVID-19 pandemic. We therefore constructed multiple robust linear regression models that regressed the cluster as a dummy variable against the respective dimension of interest. We additionally computed robust regression models that also included gender, age, education, marital status, community size and presence of a mental or somatic disease to extract the conditional effect of cluster membership on the respective domain. Again, we will not only consider statistical significance as a key criterion, but also the magnitude of the effect itself. For the sake of brevity, we refrain from supplying detailed regression and subsequent marginal effect tables, but attempt to give the reader an intuition on the data by primarily using graphical summaries.

Although the analyses revealed significant differences between the three clusters on depression scores (PHQ-2), we consider them to be of little practical significance. Here, non-helpers show the highest levels in depression scores (M = 1.80) compared to helpers (M = 1.71) and help receivers and helpers (M = 1.51). On a scale from 0 to 6, we regard a mean difference of 0.29 as miniscule and practically not relevant in our case. Furthermore, such differences became even smaller after conditioning upon the above-mentioned demographic control variables. Similarly, although helpers show the most elevated levels of generalized anxiety, these mean differences of .4-.5 are relatively negligible on a scale from 0 to 21. In line with this, we also find no or only miniscule effects on distress.

On the other side, we find that individuals in the non-helpers cluster show the lowest levels of COVID-19-related fear (see figure S4). Here, the mean differences of 0.99 (for non-helpers vs. help-receivers and helpers) and .7 (for non-helpers vs. helpers) are considerable for a Likert-scale that ranges from 1 to 7. This effect remains almost identical when including the sociodemographic covariates into the regression model. Similarly, individuals from the non-helpers cluster show lower levels of safety behavior (see figure S5, panel A). With mean differences of about 1 (for the differences between non-helpers and helpers, as well as non-helpers and help receivers and helpers on adherent safety behavior) on an average of a Likert-scale from 1 to 7, we consider this difference also practically relevant. An almost equal pattern occurs when performing the same analyses on dysfunctional safety behavior, although the mean differences are not as pronounced (see figure S5, panel B). Indeed, non-helpers also show lower levels of trust in governmental interventions to fight COVID-19. Here again, the mean differences are somewhat smaller (e.g. 0.39 for non-helpers vs. helpers on an averaged Likert-scale from 1 to 7). Differences between clusters in terms of the subjective level of information, however, remain negligible. Again, all these findings persist even after controlling for relevant sociodemographic factors.

The most prominent differences, however, are measured in the subjective risk perceptions regarding an occurrence of a COVID-19-contraction and the respective consequences ($\%\text{-}Risk_{infection}$, $\%\text{-}Risk_{symptoms}$, $\%\text{-}Risk_{severe}$, $\%\text{-Risk}_{\text{dying}}$, $\%\text{-}Risk_{transmit}$). Generally, it appears that non-helpers consider the risk of exposure to COVID-19 lower than individuals in the other clusters (see figure S6). With average differences of 7-10% (on a scale from 0% to 100%) for the risk assessment of a COVID-19 contraction for oneself and others, we consider this result quite meaningful. These patterns also emerge when controlling for the relevant sociodemographic variables. Concerning the subjective risk of developing symptoms, suffering from a severe course or dying in case of an infection, non-helpers also exhibit lower average subjective risk assessments than the other two clusters, although here, some differences are smaller and become less pronounced when controlling for gender, age, education, marital status, community size and presence of mental or somatic diseases.

| Parameter | Model 1 on item "More people offered help to me." | | | | | | Model 2 on item "I have offered more help to other people." | | | | | |
|---|---|---|---|---|---|---|---|---|---|---|---|---|
| | Coefficient | SE | 95%-CI (lower bound) | 95%-CI (upper bound) | t | p | Coefficient | SE | 95%-CI (lower bound) | 95%-CI (upper bound) | t | p |
| (Intercept) | 0.854 | 0.070 | 0.717 | 0.991 | 12.182 | 0.000 | 2.022 | 0.082 | 1.860 | 2.183 | 24.567 | 0.000 |
| Generalized Anxiety (GAD-7) | 0.003 | 0.003 | -0.004 | 0.010 | 0.872 | 0.383 | 0.022 | 0.004 | 0.014 | 0.030 | 5.463 | 0.000 |
| Depression (PHQ-2) | -0.048 | 0.009 | -0.066 | -0.030 | -5.172 | 0.000 | -0.116 | 0.011 | -0.137 | -0.095 | -10.776 | 0.000 |
| Adherent Safety Behavior | 0.116 | 0.009 | 0.099 | 0.134 | 13.087 | 0.000 | 0.301 | 0.010 | 0.281 | 0.322 | 28.873 | 0.000 |
| Dysfunctional Safety Behavior | 0.147 | 0.009 | 0.130 | 0.164 | 16.673 | 0.000 | 0.066 | 0.010 | 0.045 | 0.086 | 6.346 | 0.000 |
| Distress | -0.004 | 0.005 | -0.014 | 0.005 | -0.908 | 0.364 | 0.014 | 0.006 | 0.003 | 0.025 | 2.480 | 0.013 |
| Trust in governmental interventions in response to COVID-19 | 0.075 | 0.008 | 0.058 | 0.091 | 8.880 | 0.000 | 0.078 | 0.010 | 0.059 | 0.097 | 7.900 | 0.000 |
| Subjective level of information concerning COVID-19 | -0.045 | 0.010 | -0.064 | -0.026 | -4.544 | 0.000 | -0.025 | 0.012 | -0.048 | -0.003 | -2.190 | 0.029 |
| Fear of COVID-19 | 0.016 | 0.007 | 0.001 | 0.030 | 2.151 | 0.031 | -0.020 | 0.009 | -0.037 | -0.003 | -2.300 | 0.021 |
| %-Risk$_{infection}$ | 0.004 | 0.001 | 0.003 | 0.005 | 7.583 | 0.000 | 0.003 | 0.001 | 0.002 | 0.004 | 4.984 | 0.000 |
| %-Risk$_{symptoms}$ | 0.001 | 0.001 | 0.000 | 0.002 | 1.218 | 0.223 | -0.003 | 0.001 | -0.004 | -0.001 | -4.206 | 0.000 |
| %-Risk$_{severe}$ | 0.001 | 0.001 | -0.001 | 0.002 | 0.998 | 0.318 | -0.002 | 0.001 | -0.003 | 0.000 | -1.812 | 0.070 |
| %-Risk$_{dying}$ | 0.001 | 0.001 | -0.001 | 0.002 | 0.756 | 0.449 | -0.003 | 0.001 | -0.005 | -0.001 | -3.473 | 0.001 |
| %-Risk$_{transmit}$ | -0.002 | 0.001 | -0.003 | -0.001 | -3.339 | 0.001 | 0.004 | 0.001 | 0.003 | 0.005 | 6.183 | 0.000 |
| Age: 25-34 (ref: 18-24) | 0.145 | 0.037 | 0.072 | 0.217 | 3.900 | 0.000 | 0.048 | 0.044 | -0.037 | 0.133 | 1.104 | 0.270 |
| Age: 35-44 (ref: 18-24) | 0.198 | 0.040 | 0.119 | 0.277 | 4.933 | 0.000 | 0.149 | 0.047 | 0.057 | 0.241 | 3.167 | 0.002 |
| Age: 45-54 (ref: 18-24) | 0.136 | 0.043 | 0.052 | 0.219 | 3.190 | 0.001 | 0.220 | 0.050 | 0.122 | 0.318 | 4.397 | 0.000 |
| Age: 55-64 (ref: 18-24) | 0.217 | 0.047 | 0.125 | 0.308 | 4.629 | 0.000 | 0.118 | 0.055 | 0.010 | 0.225 | 2.144 | 0.032 |
| Age: 65-74 (ref: 18-24) | 0.899 | 0.065 | 0.771 | 1.027 | 13.769 | 0.000 | -0.104 | 0.077 | -0.255 | 0.046 | -1.363 | 0.173 |
| Age: > 75 (ref: 18-24) | 1.573 | 0.135 | 1.307 | 1.838 | 11.625 | 0.000 | -0.710 | 0.159 | -1.022 | -0.399 | -4.475 | 0.000 |
| Gender: Male (ref: Female) | -0.076 | 0.024 | -0.123 | -0.029 | -3.157 | 0.002 | -0.284 | 0.028 | -0.339 | -0.229 | -10.101 | 0.000 |
| Gender: Diverse (ref: Female) | 0.110 | 0.159 | -0.202 | 0.422 | 0.689 | 0.491 | 0.110 | 0.187 | -0.256 | 0.476 | 0.589 | 0.556 |
| Marital status: Married (ref: single) | -0.118 | 0.030 | -0.177 | -0.059 | -3.942 | 0.000 | 0.086 | 0.035 | 0.017 | 0.155 | 2.435 | 0.015 |
| Marital status: In partnership (ref: single) | -0.097 | 0.031 | -0.158 | -0.037 | -3.148 | 0.002 | 0.086 | 0.036 | 0.015 | 0.157 | 2.371 | 0.018 |
| Marital status: Divorced (ref: single) | 0.053 | 0.051 | -0.048 | 0.153 | 1.027 | 0.304 | 0.046 | 0.060 | -0.071 | 0.164 | 0.770 | 0.441 |
| Marital status: Widowed (ref: single) | 0.163 | 0.099 | -0.030 | 0.357 | 1.658 | 0.097 | 0.137 | 0.116 | -0.090 | 0.364 | 1.183 | 0.237 |
| Marital status: Others (ref: single) | 0.030 | 0.120 | -0.204 | 0.265 | 0.253 | 0.800 | 0.003 | 0.140 | -0.272 | 0.278 | 0.022 | 0.983 |
| Education: College degree (ref: University degree) | 0.099 | 0.027 | 0.047 | 0.152 | 3.740 | 0.000 | 0.135 | 0.031 | 0.074 | 0.196 | 4.328 | 0.000 |
| Education: Secondary degree Realschule (ref: University degree) | 0.110 | 0.030 | 0.051 | 0.169 | 3.672 | 0.000 | 0.108 | 0.035 | 0.039 | 0.177 | 3.071 | 0.002 |
| Education: Secondary degree Hauptschule (ref: University degree) | 0.185 | 0.052 | 0.082 | 0.288 | 3.532 | 0.000 | -0.072 | 0.062 | -0.193 | 0.048 | -1.175 | 0.240 |
| Education: No degree (ref: University degree) | 0.570 | 0.179 | 0.220 | 0.921 | 3.189 | 0.001 | 0.067 | 0.210 | -0.344 | 0.479 | 0.321 | 0.748 |
| Education: Other (ref: University degree) | 0.255 | 0.089 | 0.080 | 0.430 | 2.861 | 0.004 | 0.115 | 0.105 | -0.091 | 0.320 | 1.094 | 0.274 |
| Size of community: Medium-sized (ref: Metropolis) | -0.012 | 0.027 | -0.065 | 0.041 | -0.454 | 0.650 | 0.065 | 0.032 | 0.003 | 0.127 | 2.053 | 0.040 |
| Size of community: Small (ref: Metropolis) | 0.016 | 0.033 | -0.049 | 0.080 | 0.469 | 0.639 | -0.050 | 0.039 | -0.126 | 0.027 | -1.277 | 0.201 |
| Size of community: Rural area (ref: Metropolis) | 0.023 | 0.033 | -0.041 | 0.087 | 0.708 | 0.479 | 0.079 | 0.038 | 0.005 | 0.154 | 2.081 | 0.037 |
| Diagnosis of a mental disorder (ref: no) | -0.003 | 0.033 | -0.068 | 0.061 | -0.102 | 0.919 | -0.027 | 0.039 | -0.103 | 0.049 | -0.703 | 0.482 |

**Supplementary Table S1: Regression coefficents**

| | Model 1 on item "More people offered help to me." | | | | Model 2 on item "I have offered more help to other people." | | | |
|---|---|---|---|---|---|---|---|---|
| | Df | Sum of Squares | F-Value | p | Df | Sum.Sq | F-Value | p |
| Generalized Anxiety (GAD-7) | 1.000 | 59.315 | 19.237 | 0.000 | 1.000 | 4.645 | 2.075 | 0.150 |
| Depression (PHQ-2) | 1.000 | 1087.420 | 352.679 | 0.000 | 1.000 | 252.383 | 112.758 | 0.000 |
| Adherent Safety Behavior | 1.000 | 6385.923 | 2071.120 | 0.000 | 1.000 | 2227.866 | 995.349 | 0.000 |
| Dysfunctional Safety Behavior | 1.000 | 70.704 | 22.931 | 0.000 | 1.000 | 800.571 | 357.673 | 0.000 |
| Distress | 1.000 | 40.681 | 13.194 | 0.000 | 1.000 | 6.123 | 2.736 | 0.098 |
| Trust in governmental interventions in response to COVID-19 | 1.000 | 176.269 | 57.169 | 0.000 | 1.000 | 170.960 | 76.380 | 0.000 |
| Subjective level of information concerning COVID-19 | 1.000 | 4.081 | 1.324 | 0.250 | 1.000 | 47.134 | 21.058 | 0.000 |
| Fear of COVID-19 | 1.000 | 17.908 | 5.808 | 0.016 | 1.000 | 46.888 | 20.948 | 0.000 |
| %-Risk$_{infection}$ | 1.000 | 332.140 | 107.722 | 0.000 | 1.000 | 73.986 | 33.055 | 0.000 |
| %-Risk$_{symptoms}$ | 1.000 | 146.470 | 47.504 | 0.000 | 1.000 | 27.962 | 12.493 | 0.000 |
| %-Risk$_{severe}$ | 1.000 | 95.074 | 30.835 | 0.000 | 1.000 | 107.708 | 48.121 | 0.000 |
| %-Risk$_{dying}$ | 1.000 | 55.038 | 17.850 | 0.000 | 1.000 | 27.057 | 12.088 | 0.001 |
| %-Risk$_{transmit}$ | 1.000 | 114.069 | 36.996 | 0.000 | 1.000 | 77.353 | 34.559 | 0.000 |
| Age: 25-34 (ref: 18-24) | 6.000 | 260.905 | 14.103 | 0.000 | 6.000 | 702.934 | 52.342 | 0.000 |
| Gender | 2.000 | 343.563 | 55.713 | 0.000 | 2.000 | 27.388 | 6.118 | 0.002 |
| Marital status | 5.000 | 28.368 | 1.840 | 0.101 | 5.000 | 81.243 | 7.259 | 0.000 |
| Education | 5.000 | 86.983 | 5.642 | 0.000 | 5.000 | 86.452 | 7.725 | 0.000 |
| Community Size | 3.000 | 36.490 | 3.945 | 0.008 | 3.000 | 2.708 | 0.403 | 0.751 |
| Diagnosis of a mental disorder | 1.000 | 1.523 | 0.494 | 0.482 | 1.000 | 0.023 | 0.010 | 0.919 |
| | 19941.000 | 61484.445 | | | 19941.000 | 44633.469 | | |

**Supplementary Table S2: ANOVA tests with received and demonstrated solidarity as dependent variable**

| contrast | estimate | SE | df | t.ratio | p.value |
|---|---|---|---|---|---|
| 18-24 years old - 25-34 years old | -0.144738515 | 0.037113732 | 19941 | -3.899864224 | 0.001865998 |
| 18-24 years old - 35-44 years old | -0.197937435 | 0.040128403 | 19941 | -4.932601844 | 1.68954E-05 |
| 18-24 years old - 45-54 years old | -0.135902628 | 0.042599913 | 19941 | -3.190209041 | 0.024039154 |
| 18-24 years old - 55-64 years old | -0.216509949 | 0.04677592 | 19941 | -4.628662601 | 7.57214E-05 |
| 18-24 years old - 65-75 years old | -0.898884357 | 0.065284769 | 19941 | -13.76866876 | 0 |
| 18-24 years old - 75+ years old | -1.572582938 | 0.13527211 | 19941 | -11.62533014 | 0 |
| 25-34 years old - 35-44 years old | -0.05319892 | 0.03227074 | 19941 | -1.648518755 | 0.650555042 |
| 25-34 years old - 45-54 years old | 0.008835887 | 0.034829984 | 19941 | 0.253686217 | 0.999978169 |
| 25-34 years old - 55-64 years old | -0.071771434 | 0.039465349 | 19941 | -1.818593668 | 0.535343812 |
| 25-34 years old - 65-75 years old | -0.754145842 | 0.059437475 | 19941 | -12.68805317 | 0 |
| 25-34 years old - 75+ years old | -1.427844423 | 0.132526053 | 19941 | -10.77406586 | 0 |
| 35-44 years old - 45-54 years old | 0.062034807 | 0.033402384 | 19941 | 1.857196983 | 0.50910304 |
| 35-44 years old - 55-64 years old | -0.018572514 | 0.037538377 | 19941 | -0.494760723 | 0.998928971 |
| 35-44 years old - 65-75 years old | -0.700946922 | 0.057573637 | 19941 | -12.17478973 | 0 |
| 35-44 years old - 75+ years old | -1.374645503 | 0.131578901 | 19941 | -10.44730952 | 0 |
| 45-54 years old - 55-64 years old | -0.080607321 | 0.03768521 | 19941 | -2.138964372 | 0.329647644 |
| 45-54 years old - 65-75 years old | -0.762981729 | 0.057186498 | 19941 | -13.34199075 | 0 |
| 45-54 years old - 75+ years old | -1.43668031 | 0.131305467 | 19941 | -10.94151171 | 0 |
| 55-64 years old - 65-75 years old | -0.682374408 | 0.057811112 | 19941 | -11.80351637 | 0 |
| 55-64 years old - 75+ years old | -1.356072988 | 0.131309128 | 19941 | -10.32733225 | 0 |
| 65-75 years old - 75+ years old | -0.67369858 | 0.136493145 | 19941 | -4.935768619 | 1.66251E-05 |

**Supplementary Table S3. Marginal effects / contrasts for age on received solidarity. P-Values are Tukey-corrected.**

| contrast | estimate | SE | df | t.ratio | p.value |
|---|---|---|---|---|---|
| College degree - Hauptschule | -0.085819259 | 0.053045213 | 19941 | -1.617851157 | 0.586647129 |
| College degree - No degree | -0.470877196 | 0.178621635 | 19941 | -2.636171121 | 0.088744955 |
| College degree - Other | -0.155936668 | 0.08960425 | 19941 | -1.740282046 | 0.50494608 |
| College degree - Realschule | -0.010454083 | 0.030659315 | 19941 | -0.340975743 | 0.999394722 |
| College degree - University degree | 0.099498142 | 0.026605929 | 19941 | 3.739698143 | 0.002544432 |
| Hauptschule - No degree | -0.385057937 | 0.183736458 | 19941 | -2.09570785 | 0.289436982 |
| Hauptschule - Other | -0.070117409 | 0.100025405 | 19941 | -0.700996007 | 0.981874348 |
| Hauptschule - Realschule | 0.075365176 | 0.05375001 | 19941 | 1.402142552 | 0.725813481 |
| Hauptschule - University degree | 0.185317401 | 0.052470618 | 19941 | 3.531831885 | 0.005535359 |
| No degree - Other | 0.314940528 | 0.197534348 | 19941 | 1.594358306 | 0.602305473 |
| No degree - Realschule | 0.460423113 | 0.178992463 | 19941 | 2.572304474 | 0.104145468 |
| No degree - University degree | 0.570375338 | 0.178845214 | 19941 | 3.1892122 | 0.017915129 |
| Other - Realschule | 0.145482585 | 0.090454262 | 19941 | 1.608355235 | 0.592982451 |
| Other - University degree | 0.25543481 | 0.089274519 | 19941 | 2.861228638 | 0.048436416 |
| Realschule - University degree | 0.109952225 | 0.029946763 | 19941 | 3.671589647 | 0.003299748 |

**Supplementary Table S4. Marginal effects / contrasts for education on perceived solidarity. P-Values are Tukey-corrected.**

| contrast | estimate | SE | df | t.ratio | p.value |
|---|---|---|---|---|---|
| Divorced - In partnership | 0.149887508 | 0.051779217 | 19941 | 2.894742661 | 0.04401967 |
| Divorced - Married | 0.170606601 | 0.047086739 | 19941 | 3.623240974 | 0.003956127 |
| Divorced - Other | 0.022300395 | 0.126582688 | 19941 | 0.176172551 | 0.999976639 |
| Divorced - Single | 0.052536506 | 0.051132652 | 19941 | 1.027455137 | 0.908870421 |
| Divorced - Widowed | -0.110938081 | 0.103593773 | 19941 | -1.070895264 | 0.89303047 |
| In partnership - Married | 0.020719093 | 0.030817842 | 19941 | 0.672308365 | 0.984977062 |
| In partnership - Other | -0.127587113 | 0.120338974 | 19941 | -1.06023102 | 0.897060746 |
| In partnership - Single | -0.097351003 | 0.030924457 | 19941 | -3.148026283 | 0.02044307 |
| In partnership - Widowed | -0.260825589 | 0.098923864 | 19941 | -2.636629604 | 0.088641372 |
| Married - Other | -0.148306206 | 0.119798472 | 19941 | -1.237964089 | 0.818196368 |
| Married - Single | -0.118070095 | 0.029953093 | 19941 | -3.941833149 | 0.001141905 |
| Married - Widowed | -0.281544682 | 0.09600547 | 19941 | -2.93259001 | 0.039448219 |
| Other - Single | 0.030236111 | 0.119532366 | 19941 | 0.252953335 | 0.999860016 |
| Other - Widowed | -0.133238476 | 0.151538644 | 19941 | -0.879237619 | 0.951554336 |
| Single - Widowed | -0.163474587 | 0.098600069 | 19941 | -1.657956109 | 0.559838179 |

**Supplementary Table S5. Marginal effects / contrasts for marital status on perceived solidarity. P-Values are Tukey-corrected.**

| contrast | estimate | SE | df | t.ratio | p.value |
|---|---|---|---|---|---|
| 18-24 years old - 25-34 years old | -0.144738515 | 0.037113732 | 19941 | -3.899864224 | 0.001865998 |
| 18-24 years old - 35-44 years old | -0.197937435 | 0.040128403 | 19941 | -4.932601844 | 1.68954E-05 |
| 18-24 years old - 45-54 years old | -0.135902628 | 0.042599913 | 19941 | -3.190209041 | 0.024039154 |
| 18-24 years old - 55-64 years old | -0.216509949 | 0.04677592 | 19941 | -4.628662601 | 7.57214E-05 |
| 18-24 years old - 65-75 years old | -0.898884357 | 0.065284769 | 19941 | -13.76866876 | 0 |
| 18-24 years old - 75+ years old | -1.572582938 | 0.13527211 | 19941 | -11.62533014 | 0 |
| 25-34 years old - 35-44 years old | -0.05319892 | 0.03227074 | 19941 | -1.648518755 | 0.650555042 |
| 25-34 years old - 45-54 years old | 0.008835887 | 0.034829984 | 19941 | 0.253686217 | 0.999978169 |
| 25-34 years old - 55-64 years old | -0.071771434 | 0.039465349 | 19941 | -1.818593668 | 0.535343812 |
| 25-34 years old - 65-75 years old | -0.754145842 | 0.059437475 | 19941 | -12.68805317 | 0 |
| 25-34 years old - 75+ years old | -1.427844423 | 0.132526053 | 19941 | -10.77406586 | 0 |
| 35-44 years old - 45-54 years old | 0.062034807 | 0.033402384 | 19941 | 1.857196983 | 0.50910304 |
| 35-44 years old - 55-64 years old | -0.018572514 | 0.037538377 | 19941 | -0.494760723 | 0.998928971 |
| 35-44 years old - 65-75 years old | -0.700946922 | 0.057573637 | 19941 | -12.17478973 | 0 |
| 35-44 years old - 75+ years old | -1.374645503 | 0.131578901 | 19941 | -10.44730952 | 0 |
| 45-54 years old - 55-64 years old | -0.080607321 | 0.03768521 | 19941 | -2.138964372 | 0.329647644 |
| 45-54 years old - 65-75 years old | -0.762981729 | 0.057186498 | 19941 | -13.34199075 | 0 |
| 45-54 years old - 75+ years old | -1.43668031 | 0.131305467 | 19941 | -10.94151171 | 0 |
| 55-64 years old - 65-75 years old | -0.682374408 | 0.057811112 | 19941 | -11.80351637 | 0 |
| 55-64 years old - 75+ years old | -1.356072988 | 0.131309128 | 19941 | -10.32733225 | 0 |
| 65-75 years old - 75+ years old | -0.67369858 | 0.136493145 | 19941 | -4.935768619 | 1.66251E-05 |

**Supplementary Table S6: Marginal effects / contrasts for age on demonstrated solidarity. P-Values are Tukey-corrected.**

| contrast | estimate | SE | df | t.ratio | p.value |
|---|---|---|---|---|---|
| Diverse - Female | 0.10968287 | 0.159088609 | 19941 | 0.689445151 | 0.769664514 |
| Diverse - Male | 0.185390533 | 0.15953495 | 19941 | 1.16206846 | 0.475970339 |
| Female - Male | 0.075707663 | 0.023981534 | 19941 | 3.15691499 | 0.004551244 |

**Supplementary Table S7: Marginal effects / contrasts for gender on demonstrated solidarity. P-Values are Tukey-corrected.**

| Parameter | Model 1 on item "More people offered help to me." Coefficient | SE | 95%-CI (lower bound) | 95%-CI (upper bound) | t | p | Model 2 on item "I have offered more help to other people." Coefficient | SE | 95%-CI (lower bound) | 95%-CI (upper bound) | t | p |
|---|---|---|---|---|---|---|---|---|---|---|---|---|
| (Intercept) | 0.854 | 0.067 | 0.723 | 0.985 | 12.779 | 0.000 | 2.022 | 0.086 | 1.853 | 2.191 | 23.441 | 0.000 |
| Generalized Anxiety (GAD-7) | 0.003 | 0.003 | -0.004 | 0.010 | 0.877 | 0.381 | 0.022 | 0.004 | 0.014 | 0.030 | 5.255 | 0.000 |
| Depression (PHQ-8) | -0.048 | 0.009 | -0.065 | -0.030 | -5.244 | 0.000 | -0.116 | 0.011 | -0.138 | -0.094 | -10.325 | 0.000 |
| Adherent Safety Behavior | 0.116 | 0.008 | 0.100 | 0.133 | 13.749 | 0.000 | 0.301 | 0.011 | 0.279 | 0.323 | 27.054 | 0.000 |
| Dysfunctional Safety Behavior | 0.147 | 0.009 | 0.128 | 0.165 | 15.593 | 0.000 | 0.066 | 0.011 | 0.045 | 0.086 | 6.201 | 0.000 |
| Distress | -0.004 | 0.005 | -0.014 | 0.005 | -0.909 | 0.364 | 0.014 | 0.006 | 0.003 | 0.025 | 2.403 | 0.016 |
| Trust in governmental interventions in response to COVID-19 | 0.075 | 0.008 | 0.058 | 0.091 | 8.904 | 0.000 | 0.078 | 0.010 | 0.058 | 0.098 | 7.549 | 0.000 |
| Subjective level of information concerning COVID-19 | -0.045 | 0.009 | -0.063 | -0.027 | -4.828 | 0.000 | -0.025 | 0.012 | -0.050 | -0.001 | -2.049 | 0.040 |
| Fear of COVID-19 | 0.016 | 0.007 | 0.002 | 0.030 | 2.180 | 0.029 | -0.020 | 0.009 | -0.037 | -0.002 | -2.192 | 0.028 |
| %-Risk$_{infection}$ | 0.004 | 0.001 | 0.003 | 0.005 | 7.266 | 0.000 | 0.003 | 0.001 | 0.002 | 0.004 | 4.766 | 0.000 |
| %-Risk$_{symptoms}$ | 0.001 | 0.001 | 0.000 | 0.002 | 1.198 | 0.231 | -0.003 | 0.001 | -0.004 | -0.001 | -4.080 | 0.000 |
| %-Risk$_{severe}$ | 0.001 | 0.001 | -0.001 | 0.002 | 0.931 | 0.352 | -0.002 | 0.001 | -0.003 | 0.000 | -1.756 | 0.079 |
| %-Risk$_{dying}$ | 0.001 | 0.001 | -0.001 | 0.002 | 0.674 | 0.500 | -0.003 | 0.001 | -0.005 | -0.001 | -3.345 | 0.001 |
| %-Risk$_{transmit}$ | -0.002 | 0.001 | -0.003 | -0.001 | -3.210 | 0.001 | 0.004 | 0.001 | 0.002 | 0.005 | 5.963 | 0.000 |
| Age: 25-34 (ref: 18-24) | 0.145 | 0.035 | 0.075 | 0.214 | 4.079 | 0.000 | 0.048 | 0.044 | -0.038 | 0.134 | 1.092 | 0.275 |
| Age: 35-44 (ref: 18-24) | 0.198 | 0.039 | 0.121 | 0.274 | 5.071 | 0.000 | 0.149 | 0.047 | 0.057 | 0.242 | 3.156 | 0.002 |
| Age: 45-54 (ref: 18-24) | 0.136 | 0.042 | 0.054 | 0.218 | 3.259 | 0.001 | 0.220 | 0.050 | 0.122 | 0.318 | 4.397 | 0.000 |
| Age: 55-64 (ref: 18-24) | 0.217 | 0.047 | 0.125 | 0.308 | 4.649 | 0.000 | 0.118 | 0.056 | 0.008 | 0.228 | 2.102 | 0.036 |
| Age: 65-74 (ref: 18-24) | 0.899 | 0.074 | 0.754 | 1.043 | 12.191 | 0.000 | -0.104 | 0.078 | -0.257 | 0.048 | -1.341 | 0.180 |
| Age: > 75 (ref: 18-24) | 1.573 | 0.188 | 1.203 | 1.942 | 8.351 | 0.000 | -0.710 | 0.165 | -1.034 | -0.387 | -4.310 | 0.000 |
| Gender: Male (ref: Female) | -0.076 | 0.023 | -0.121 | -0.030 | -3.247 | 0.001 | -0.284 | 0.029 | -0.341 | -0.228 | -9.915 | 0.000 |
| Gender: Diverse (ref: Female) | 0.110 | 0.193 | -0.268 | 0.488 | 0.569 | 0.570 | 0.110 | 0.202 | -0.286 | 0.506 | 0.544 | 0.586 |
| Marital status: Married (ref: single) | -0.118 | 0.031 | -0.178 | -0.058 | -3.869 | 0.000 | 0.086 | 0.035 | 0.016 | 0.155 | 2.414 | 0.016 |
| Marital status: In partnership (ref: single) | -0.097 | 0.031 | -0.157 | -0.037 | -3.186 | 0.001 | 0.086 | 0.036 | 0.015 | 0.157 | 2.365 | 0.018 |
| Marital status: Divorced (ref: single) | 0.053 | 0.054 | -0.053 | 0.158 | 0.974 | 0.330 | 0.046 | 0.061 | -0.073 | 0.165 | 0.761 | 0.446 |
| Marital status: Widowed (ref: single) | 0.163 | 0.121 | -0.073 | 0.400 | 1.352 | 0.176 | 0.137 | 0.123 | -0.104 | 0.378 | 1.115 | 0.265 |
| Marital status: Others (ref: single) | 0.030 | 0.118 | -0.201 | 0.262 | 0.256 | 0.798 | 0.003 | 0.143 | -0.278 | 0.284 | 0.021 | 0.983 |
| Education: College degree (ref: University degree) | 0.099 | 0.027 | 0.047 | 0.152 | 3.746 | 0.000 | 0.135 | 0.031 | 0.074 | 0.196 | 4.351 | 0.000 |
| Education: Secondary degree Realschule (ref: University degree) | 0.110 | 0.030 | 0.051 | 0.169 | 3.674 | 0.000 | 0.108 | 0.035 | 0.039 | 0.177 | 3.059 | 0.002 |
| Education: Secondary degree Hauptschule (ref: University degree) | 0.185 | 0.056 | 0.076 | 0.294 | 3.336 | 0.001 | -0.072 | 0.064 | -0.198 | 0.053 | -1.128 | 0.259 |
| Education: No degree (ref: University degree) | 0.570 | 0.231 | 0.119 | 1.022 | 2.474 | 0.013 | 0.067 | 0.237 | -0.398 | 0.533 | 0.284 | 0.776 |
| Education: Other (ref: University degree) | 0.255 | 0.102 | 0.055 | 0.456 | 2.495 | 0.013 | 0.115 | 0.107 | -0.096 | 0.325 | 1.069 | 0.285 |
| Size of community: Medium-sized (ref: Metropolis) | -0.012 | 0.027 | -0.065 | 0.040 | -0.457 | 0.648 | 0.065 | 0.031 | 0.004 | 0.126 | 2.073 | 0.038 |
| Size of community: Small (ref: Metropolis) | 0.016 | 0.033 | -0.050 | 0.081 | 0.465 | 0.642 | -0.050 | 0.039 | -0.127 | 0.028 | -1.258 | 0.208 |
| Size of community: Rural area (ref: Metropolis) | 0.023 | 0.032 | -0.040 | 0.086 | 0.716 | 0.474 | 0.079 | 0.039 | 0.003 | 0.156 | 2.044 | 0.041 |
| Diagnosis of a mental disorder (ref: no) | -0.003 | 0.033 | -0.069 | 0.062 | -0.100 | 0.920 | -0.027 | 0.039 | -0.103 | 0.048 | -0.704 | 0.482 |

**Supplementary Table S8: Regression coefficients of robust regression model.**

| | Level | Help receivers and helpers | Non-helpers | Helpers | p |
|---|---|---|---|---|---|
| n | | 5,224 | 5,756 | 8,998 | |
| Age | 18-24 yrs. | 661 (12.7) | 939 (16.3) | 1475 (16.4) | <0.001 |
| | 25-34 yrs. | 1142 (21.9) | 1429 (24.8) | 2186 (24.3) | |
| | 35-44 yrs. | 1158 (22.2) | 1270 (22.1) | 2036 (22.6) | |
| | 45-54 yrs. | 987 (18.9) | 1006 (17.5) | 1815 (20.2) | |
| | 55-64 yrs. | 807 (15.4) | 811 (14.1) | 1203 (13.4) | |
| | 65-74 yrs. | 391 ( 7.5) | 254 ( 4.4) | 267 ( 3.0) | |
| | 75+ yrs. | 78 ( 1.5) | 47 ( 0.8) | 16 ( 0.2) | |
| Gender | Female | 3625 (69.4) | 3359 (58.4) | 6322 (70.3) | <0.001 |
| | Male | 1570 (30.1) | 2370 (41.2) | 2640 (29.3) | |
| | Diverse | 28 ( 0.5) | 27 ( 0.5) | 36 ( 0.4) | |
| Education | University degree | 2027 (38.8) | 2290 (39.8) | 3677 (40.9) | <0.001 |
| | High school degree | 1648 (31.5) | 1781 (30.9) | 2998 (33.3) | |
| | Realschule | 1131 (21.7) | 1275 (22.2) | 1814 (20.2) | |
| | Hauptschule | 302 ( 5.8) | 308 ( 5.4) | 359 ( 4.0) | |
| | No degree | 26 ( 0.5) | 24 ( 0.4) | 22 ( 0.2) | |
| | Other | 90 ( 1.7) | 78 ( 1.4) | 128 ( 1.4) | |
| Marital status | Single | 1513 (29.0) | 1879 (32.6) | 2605 (29.0) | <0.001 |
| | Married | 2214 (42.4) | 2255 (39.2) | 3765 (41.8) | |
| | In partnership | 985 (18.9) | 1181 (20.5) | 1974 (21.9) | |
| | Divorced | 362 ( 6.9) | 324 ( 5.6) | 497 ( 5.5) | |
| | Widowed | 99 ( 1.9) | 68 ( 1.2) | 94 ( 1.0) | |
| | Other | 51 ( 1.0) | 49 ( 0.9) | 63 ( 0.7) | |
| Community size | Metropolis | 2569 (49.2) | 2686 (46.7) | 4417 (49.1) | 0.002 |
| | Middle-sized city | 1199 (23.0) | 1336 (23.2) | 2159 (24.0) | |
| | Small town | 710 (13.6) | 855 (14.9) | 1150 (12.8) | |
| | Rural community | 746 (14.3) | 879 (15.3) | 1272 (14.1) | |
| Diagnosed somatic disease* | no | 3968 (76.0) | 4532 (78.7) | 7176 (79.8) | <0.001 |
| | yes | 1256 (24.0) | 1224 (21.3) | 1822 (20.2) | |
| Diagnosed mental disease | no | 4461 (85.4) | 4964 (86.2) | 7594 (84.4) | 0.008 |
| | yes | 763 (14.6) | 792 (13.8) | 1404 (15.6) | |

*That predisposes to a severe course in case of COVID-19-infecton

**Supplementary Table S9.** Demographic characteristics stratified across the three clusters. **The final column reports p-values of $\chi^2$-tests.**

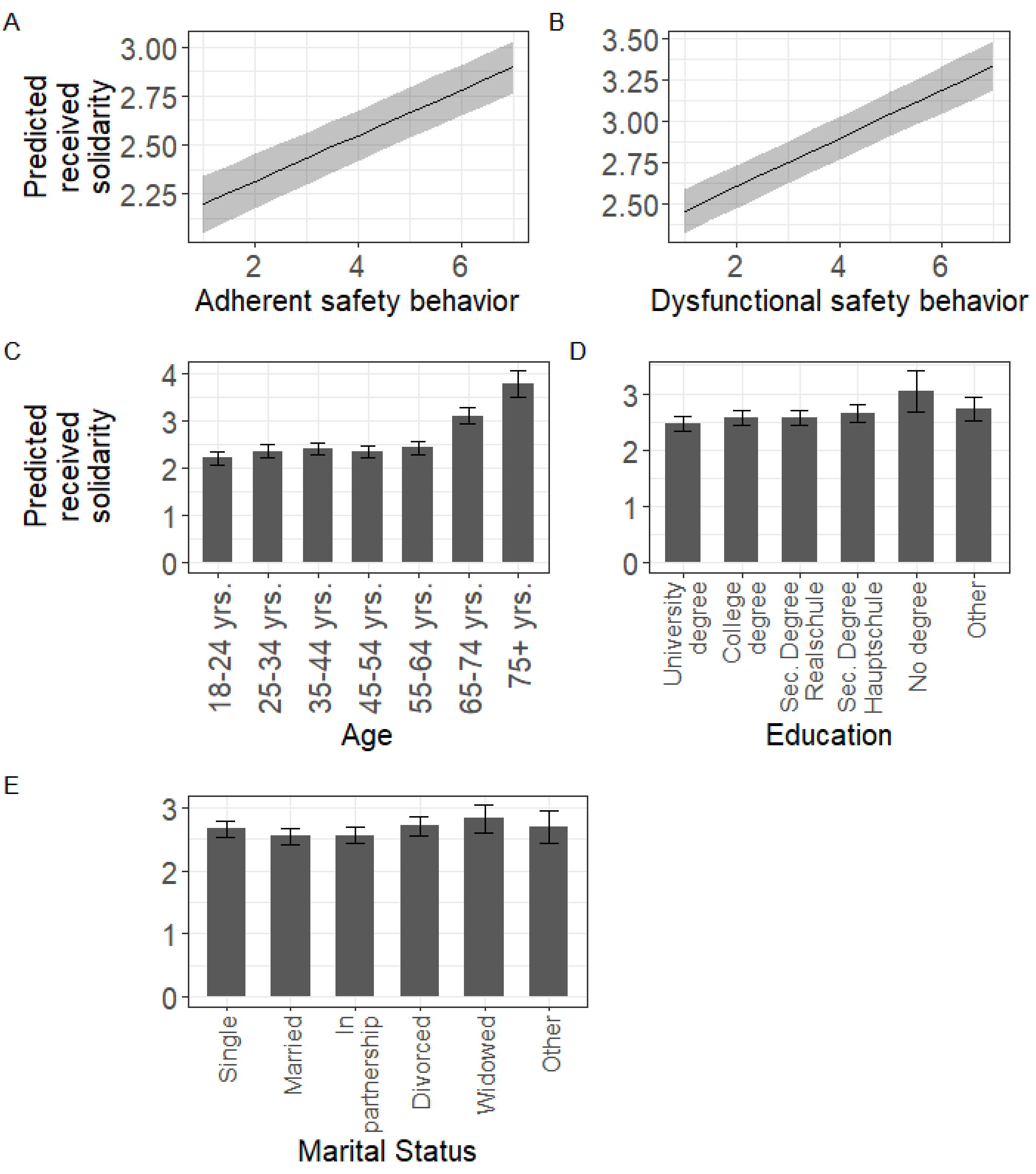


**Supplementary Figure S1.** Marginal effects of the most dominant effects of predictors regressed on received solidarity. Shaded areas and error bars represent 95%-confidence intervals.

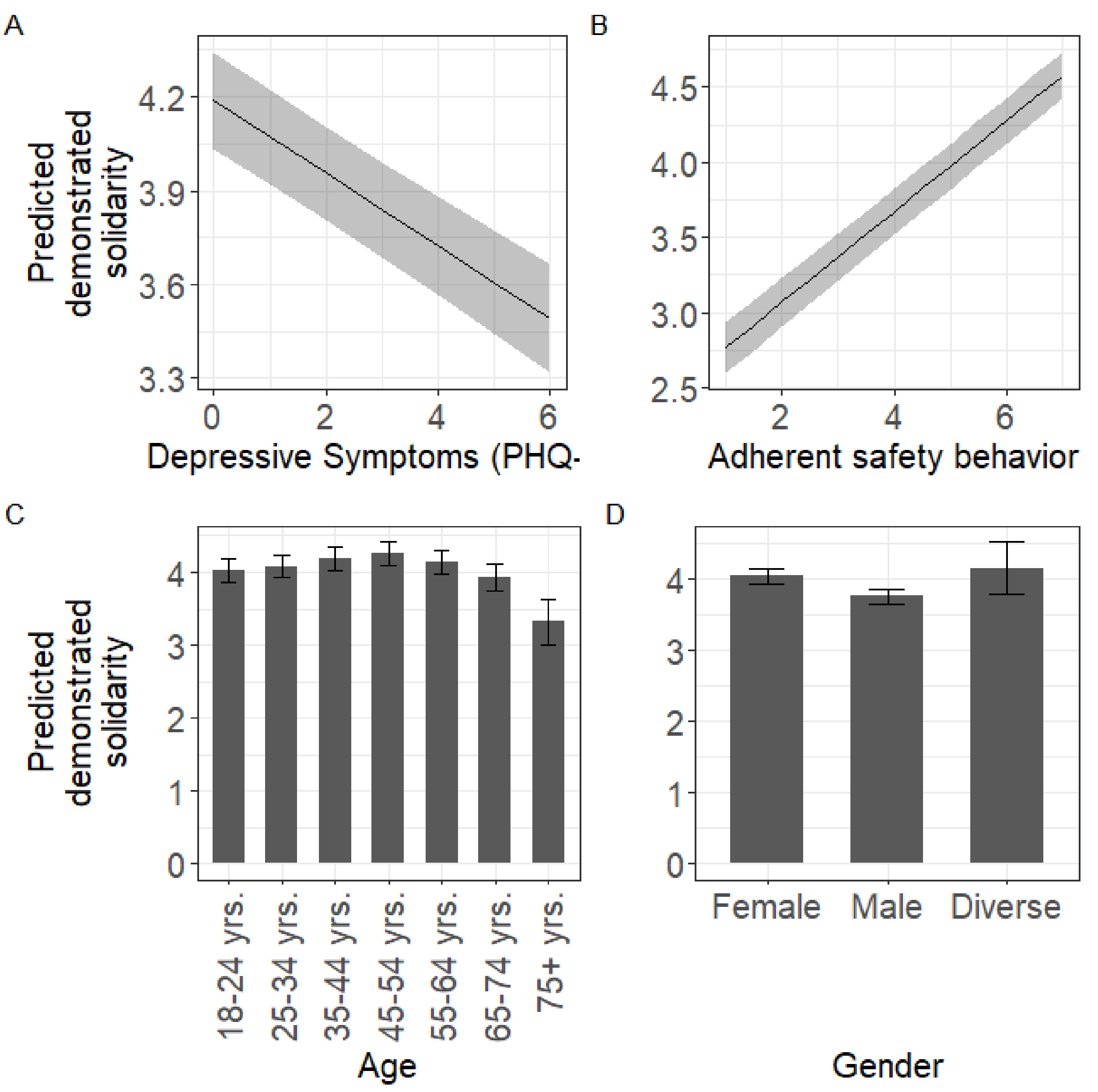


**Supplementary Figure S2.** Marginal effects of the most dominant effects of predictors regressed on demonstrated solidarity. Shaded areas and error bars represent 95%-confidence intervals.

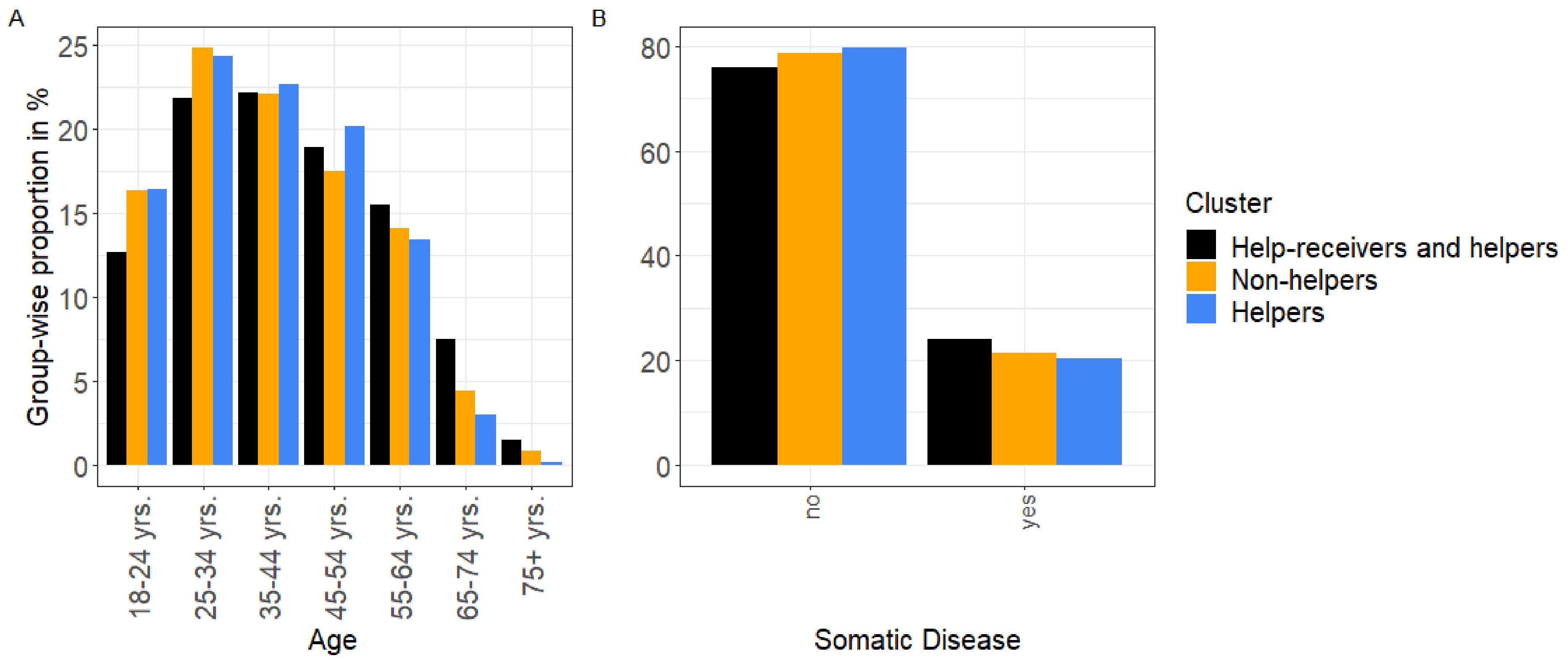


**Supplementary Figure S3. Group-wise proportions of participant characteristics.** Individuals in the help-receivers and helpers cluster are proportionally older (panel A) and slightly more likely to be diagnosed with a somatic disease that predisposes them to a more severe course of COVID-19 (panel B).

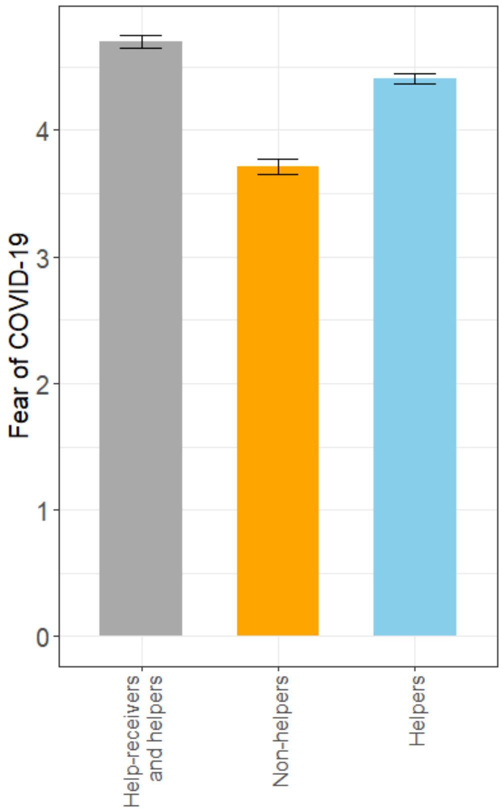


**Supplementary Figure S4.** Differences between the clusters in terms of their levels of COVID-19-related fear. Bar charts represent marginal estimated means. Error bars represent 95%-CIs.

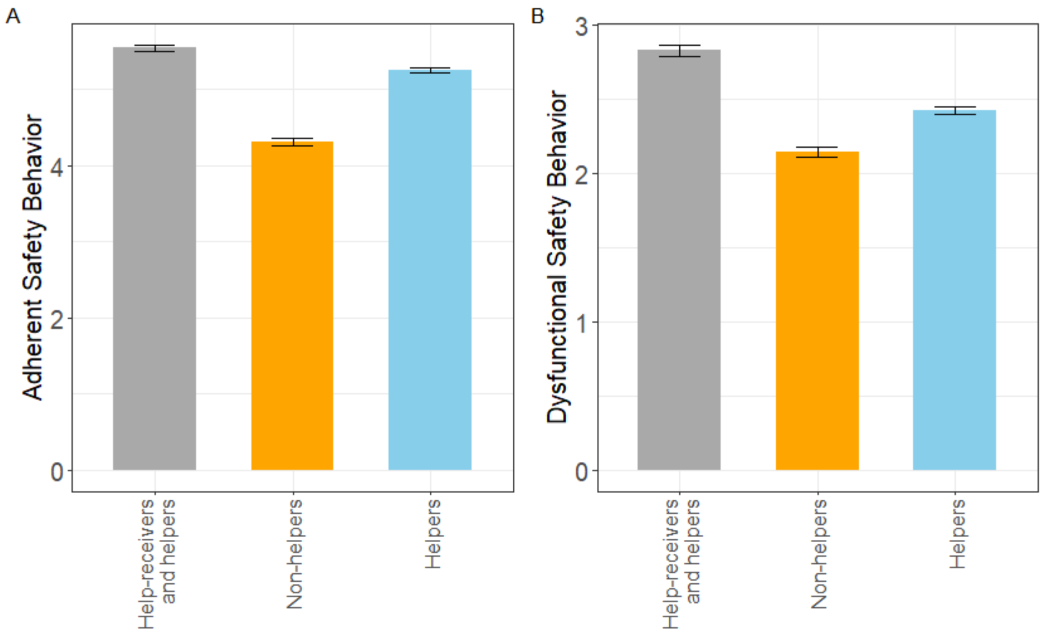


**Supplementary Figure S5.** Differences between the clusters in terms of their levels of adherent and dysfunctional safety behavior. Bar charts represent marginal estimated means. Error bars represent 95%-CIs.

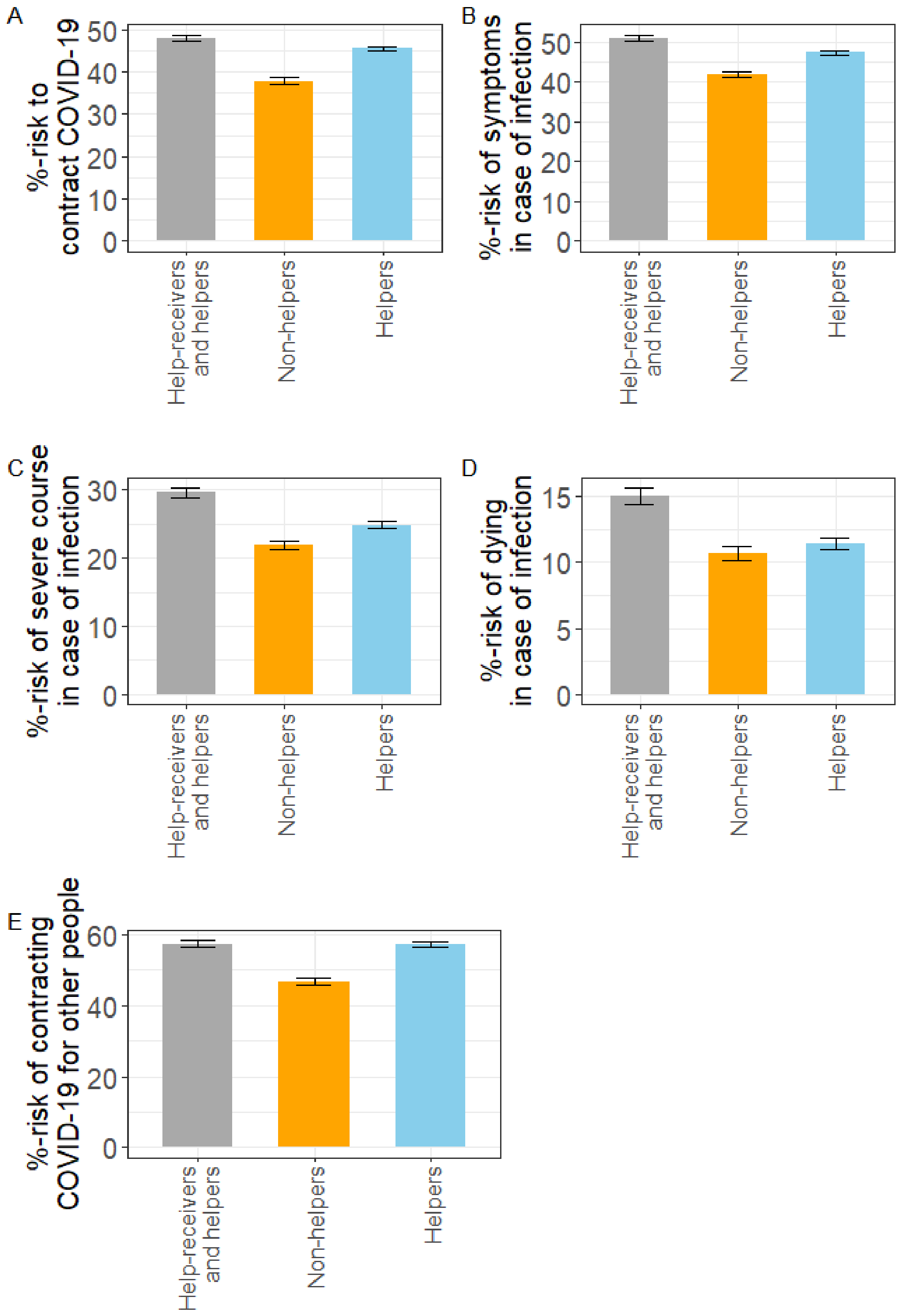
A
%-risk to contract COVID-19
B
%-risk of symptoms in case of infection
C
%-risk of severe course in case of infection
D
%-risk of dying in case of infection
E
%-risk of contracting COVID-19 for other people
Help-receivers and helpers
Non-helpers
Helpers

**Supplementary Figure S6.** Differences between the clusters in terms of their subjective risk assessments as to contracting COVID-19 ($\%$-$Risk_{infection}$), developing symptoms ($\%$-$Risk_{symptoms}$), suffering a severe course ($\%$-$Risk_{severe}$), dying from COVID-19 in case of an infection ($\%$-$Risk_{dying}$), and the risk of others to contract COVID-19 ($\%$-$Risk_{transmit}$). Bar charts represent marginal estimated means. Error bars represent 95%-CIs.
